# Age- and sex-specific spatio-temporal patterns of colorectal cancer mortality in Spain (1975-2008)

**DOI:** 10.1186/1478-7954-12-17

**Published:** 2014-07-10

**Authors:** Jaione Etxeberria, María Dolores Ugarte, Tomás Goicoa, Ana F Militino

**Affiliations:** 1 Department of Statistics and O. R., Public University of Navarre, Campus de Arrosadia, Pamplona, Navarre, Spain; 2 Consortium for Biomedical Research in Epidemiology and Public Health (CIBERESP), Madrid, Spain; 3 Research Network on Health Services in Chronic Diseases (REDISSEC), Pamplona, Spain

**Keywords:** Colorectal cancer mortality, Space-time CAR models, Disease mapping

## Abstract

In this paper, space-time patterns of colorectal cancer (CRC) mortality risks are studied by sex and age group (50-69, ≥70) in Spanish provinces during the period 1975-2008. Space-time conditional autoregressive models are used to perform the statistical analyses. A pronounced increase in mortality risk has been observed in males for both age-groups. For males between 50 and 69 years of age, trends seem to stabilize from 2001 onward. In females, trends reflect a more stable pattern during the period in both age groups. However, for the 50-69 years group, risks take an upward trend in the period 2006-2008 after the slight decline observed in the second half of the period. This study offers interesting information regarding CRC mortality distribution among different Spanish provinces that could be used to improve prevention policies and resource allocation in different regions.

## 1 Introduction

Cancer is the leading cause of death each year worldwide, and half of all deaths by cancer are due to lung, stomach, liver, colorectal, and female breast cancer [1]. About 608,000 deaths from colorectal cancer (CRC) have been estimated worldwide annually, making it the fourth most common cause of death from cancer. In the European Union, colorectal cancer is the second most common cancer. In 2008, 450,621 people suffered from this cancer and 223,268 patients (115,624 men) died [1]. CRC mortality rates vary among sex, age, and also among countries. Approximately 75% of colorectal cancer deaths occur in people older than 65 years of age [2] and in general mortality trends are falling, the decrease being generally larger in young and middle-age than in the elderly [3]. By sex, lower mortality rates are observed for females than for males, and age- and sex-specific mortality analyses indicate that mortality rates for males are comparable with those corresponding to women approximately four to eight years older [4].

Some differences in colorectal cancer mortality were also found by country. In the European Union a favorable pattern in colorectal cancer mortality for both sexes was observed in countries such as Austria, France, Finland, Ireland, Italy, Netherlands, Norway, Sweden, Switzerland, and United Kingdom from the 1990s onwards, or even earlier in Belgium, Denmark, and Germany. On the other hand, colorectal cancer mortality rates were still in an upward direction in Bulgaria, Poland, and Romania (Eastern European countries), as well as in some Mediterranean countries, such as Greece, Portugal, and Spain, between 2005 and 2007 [1,5,6]. Different populations worldwide experience various levels of colorectal cancer, and these levels change with time [7]. Geographical inequalities and temporal trends in small areas of CRC incidence [8], mortality [9,10], or even surveillance [11] have been analyzed in the literature detecting interesting differences.

**Figure 1:**
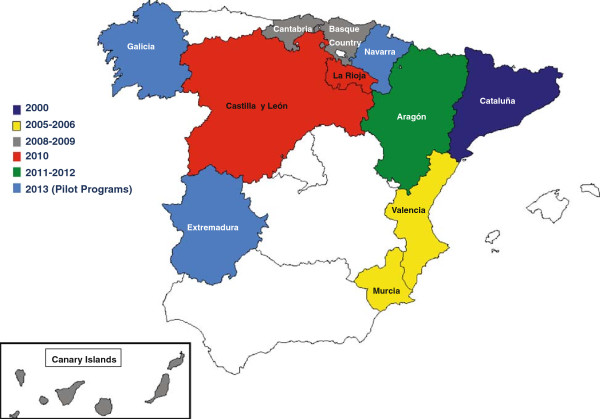
**Colorectal cancer screening programs in Spain (see **[13]**).**

The purpose of this study is to examine spatio-temporal patterns of CRC mortality relative risks by sex in Spain during the period 1975-2008. We focus on two age groups: the middle-age (50-69 years) and the elderly (≥70 years) to shed light on patterns of trends by sex, region, and age group. For the sake of explanation, Spain is organized administratively into 17 Autonomous Regions and two Autonomous Cities (Ceuta and Melilla, two enclaves located on the North-African coast). Each Autonomous Region consists of one or more provinces, for a total of 50. The Spanish National Health Service ensures access to health care to all citizens, but each Autonomous Region is responsible for its own health system. Mortality rates depend in part on survival, and therefore on advances in medical technology such as diagnostics and treatments [12] but also on access to prevention programs and medical care, which in turn depend on the specific health care system, and this may induce spatial variability in CRC mortality. Figure 1 shows the Autonomous Regions with colorectal cancer screening programs and when they were initiated [13]. In addition, Spain shows heterogeneity regarding lifestyles and socioeconomic factors.Urbanization and industrialization have not progressed at the same pace and to the same extent in all Spanish provinces, and this leads to different geographical mortality patterns for each cancer typology [14]. Colorectal cancer mortality rates in Spain showed an increase between 1985 and 2004 with an annual percent change (APC) of 2.4% in males and 0.4% in females, with a trend towards stabilization in the last few years [15]. In 2007 one out of seven cancer-related deaths was due to CRC, which makes it the second-leading cause of death from cancer in males (after lung cancer) and females (after breast cancer).

## 2 Methods

Age- and sex-specific CRC registered deaths and population data were obtained for 50 Spanish provinces (excluding Ceuta and Melilla) for the period 1975-2008 from the Spanish Statistical Office. Different revisions of the international classification of diseases (ICD) were involved in the studied period. Codes 153-154 and 159.0 (ICD-9) for colon and rectum cancer were used until 1998, and from 1998 onwards, codes C18-C21 and C26.0 (ICD-10) were considered.

### 2.1 Mortality data collection

The statistical analysis was carried out for the following four age-sex groups: males 50-69 years, females 50-69 years, males ≥70 years, and females ≥70 years. The limited number of deaths for individuals under 50 years leads us to exclude this age group for statistical analysis. Traditionally, raw measures such as the standardized mortality ratio (SMR) have been used to estimate mortality risks. However, they are highly variable in low populated areas or when the number of observed counts is small [16], and models are required to obtain reliable estimates by borrowing information from neighbouring areas in space and time. To analyze how the geographical patterns of the relative-risks (risks hereafter in the paper) evolve with time, a model with conditional autoregressive (CAR) distributions for space, a random walk of first order for time, and the corresponding space-time interactions [17,18] is employed. A description of the model is briefly provided.

Let us suppose that the area under study (Spain in this paper) is divided into small areas (here provinces) denoted by *i*=1,…,50, and data are available for different time periods *t*=1975,…,2008. Conditional on the relative risk for province *i* and time *t* in a specific age-sex group *r*_*i* *t*_, the number of deaths in each province and time period, *C*_*i* *t*_, follows a Poisson distribution with mean *μ*_*i* *t*_=*e*_*i* *t*_*r*_*i* *t*_, where *e*_*i* *t*_ is the expected number of mortality cases in this group obtained considering the Spanish population as the reference population. More precisely, the expected number of cases in each age-sex group, area *i* and time *t* is obtained as $e_{\text{it}}=\underset{k}{\sum }R_{k}n_{\text{itk}}$, where *k* denotes the five-year age-specific group within the broader age-sex category. For instance, *k*=50-54,55-59,60-64,65-69 for 50-69 years age-group. *R*_*k*_ denotes the five-year age-specific rates and *n*_*i* *t* *k*_ is the population at risk in the area *i*, period *t* and five-year age-group *k*. That is, standardization is made by age considering five-year age-groups within each larger age group analyzed here. Explicitly, the statistical model is defined as

$$\begin{array}{rrrr} C_{\text{it}}|r_{\text{it}}∼\text{Poisson}(\mu_{\text{it}}=e_{\text{it}}r_{\text{it}}), & & & \\ \log\mu_{\text{it}}=\log e_{\text{it}}+\log r_{\text{it}}. & & & \end{array}$$

If *β* is defined as an overall risk level, and *ϕ*_*i*_, *γ*_*t*_, and *δ*_*i* *t*_ denote the spatial, temporal, and spatio-temporal random effects respectively, the log-risk is modeled as

$$u_{\text{it}}=\log r_{\text{it}}=\beta +\phi_{i}+\gamma_{t}+\delta_{\text{it}},$$

where ***ϕ***, ***γ*** and ***δ*** are vectors of spatial, temporal and spatio-temporal random effects with assumed multivariate Gaussian distributions

$$\begin{array}{llll} \phi ∼N(0,\sigma_{s}^{2}D_{s}) & ; & D_{s}={\left(\lambda_{s}Q_{s}+(1-\lambda_{s})I_{s}\right)}^{-}, & \\ \gamma ∼N(\text{0},\sigma_{t}^{2}D_{t}) & ; & D_{t}=Q_{t}^{-}, & \\ \delta ∼N(\text{0},\sigma_{\text{st}}^{2}D_{\text{st}}) & ; & D_{\text{st}}=Q_{t}^{-}⊗Q_{s}^{-}. & \end{array}$$

In these expressions ^-^ represents the Moore-Penrose generalized inverse of a matrix. The spatial neighbourhood structure (provinces are neighbours if they share a common border) determines the matrix **Q**_*s*_. The *i*th diagonal element of this matrix is equal to the number of neighbours of the *i*th region. The off-diagonal entries *ij* take the value -1 if regions *i* and *j* are neighbours and 0 otherwise. The matrix **I**_*s*_ represents the identity matrix of dimension 50×50. The distribution of the spatial random effect is based on the parameterisation proposed by Leroux *et al.*[19], where *λ*_*s*_ is a spatial smoothing parameter that takes values between 0 and 1. Note that when *λ*_*s*_=0, there is no spatial variability, and when *λ*_*s*_=1, all the variability is spatial. **Q**_*t*_ is determined by the temporal neighbourhood structure. Each year has two neighbours, the previous and the following one, except the first and the last years that have only one neighbour. This definition corresponds to a random walk of first order (see [20], p. 95). The model is estimated using penalized quasi-likelihood (PQL) [21,22], which has been shown to perform well in a spatio-temporal setting [23]. R code [24] used to fit the model is available under request.

Initially, the significance of the spatio-temporal interaction effect was assessed to decide whether or not it should be introduced in the model. This is usually achieved by testing if the variance component of the spatio-temporal random effect is zero ($H_{0}:\sigma_{\text{st}}^{2}=0$). As the null hypothesis lies on the boundary of the parameter space, well known likelihood ratio tests and score tests do not follow the classical *χ*^2^ distribution [25-28]. Here, a score test and its bootstrap null distribution is used instead (see [29] for more detail). The probability of rejection has been calculated from the null distribution of the score test obtained with 300 bootstrap replicates for each of the datasets described at the beginning of this subsection. In all cases, the null hypothesis is rejected at 5% significance level. As a result the spatio-temporal interaction was included in the model. This completely structured interaction means that the temporal trend in a given region is similar to the average trend in neighbouring regions.

To show a general overview of colorectal cancer mortality throughout the period in Spain, the spatio-temporal pattern of CRC mortality risks are plotted for both males and females in the different age groups. Secondly, for a more detailed analysis, temporal trends are represented by sex and age groups (50-69 and ≥70) for each region. Confidence intervals for the risks are also given [30]. These measures help us to detect extreme risk areas. To do that, the relative risks should be interpreted as follows. The risk of Spain in the whole period is represented as a horizontal line at one. A lower bound of the confidence band above the horizontal line indicates that the CRC mortality risk in that area and year is significantly higher than the risk of the whole of Spain in the studied period. On the other hand, if the upper bound of the confidence band is below the horizontal line, the risk of that area and year is significantly lower than the risk of the whole country in the studied period. Finally, if the horizontal line is between the lower and the upper bounds of the confidence band, the risk of that area is not statistically different from the risk of Spain.

## 3 Results

**Figure 2:**
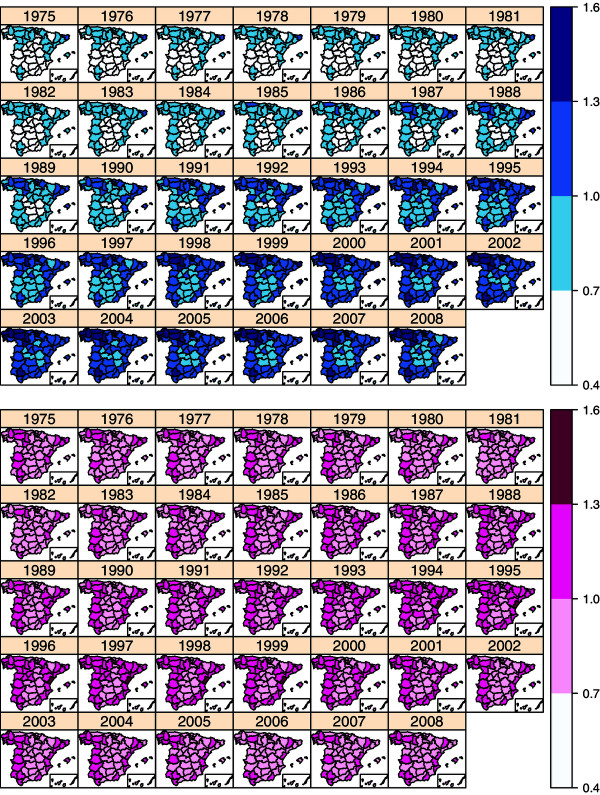
**Spatio-temporal distribution of colorectal cancer mortality risks between 1975-2008 for males (blue maps) and females (pink maps) aged between 50 and 69 years.** Note that location of the Canary Island is shown in an inset at the bottom right corner.

**Figure 3:**
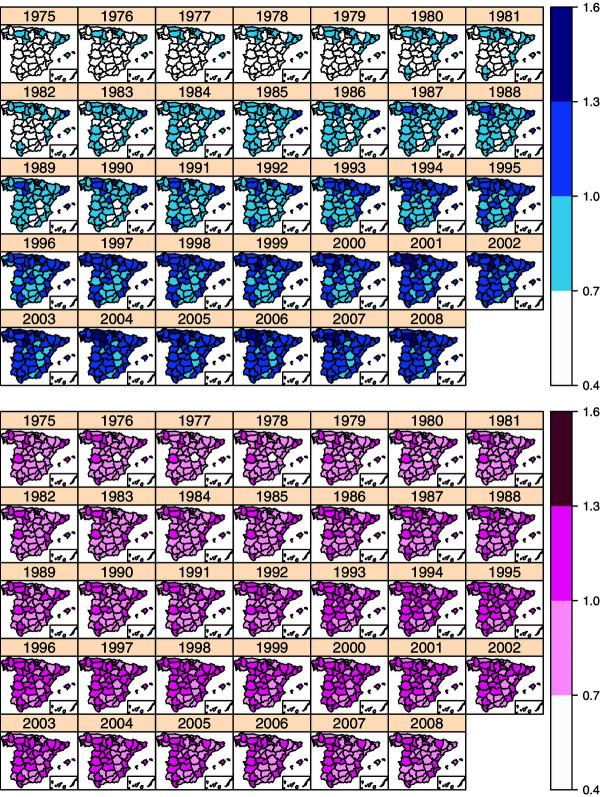
**Spatio-temporal distribution of colorectal cancer mortality risks between 1975-2008 for males (blue maps) and females (pink maps) above 69 years.** Note that location of the Canary Island is shown in an inset at the bottom right corner.

As an initial exploratory analysis, Table 1 displays the number of deaths and crude mortality rates (per 100,000 inhabitants) in the period 1975-2008 divided by tumour, sex, and age group. From the results in Table 1, mortality rates for males seem to be higher than for females, and rates also seem to increase with age.Figure 2 displays the evolution of the geographical patterns of colorectal mortality risks in Spain during the study period for males (top blue maps) and females (bottom pink maps) aged between 50 and 69 years. For males, the regions in the northwest, northeast, and southwest of Spain had the highest risk at the beginning of the period. In the 1980s, an increase in risk is observed from north to south in both the western and eastern bands of the country with the central part of the country maintaining a low risk. Then, risk increases in all regions during the 1990s and throughout the period. This is particularly relevant in some areas in the northwest (Coruña, Lugo, Pontevedra, Asturias, Cantabria, León, Palencia, Valladolid, Vizcaya, and Álava) and southwest (Sevilla) of the country. Other provinces in the northeast and eastern area also exhibit high risk (Lleida, Girona, Barcelona, Tarragona, Castellón, and Valencia). On the other hand, some provinces in the central area remain with risk lower than one (Soria, Segovia, Ávila, Toledo, Cuenca, Ciudad Real, Albacete, and Guadalajara). For females, a group of regions in the northwest, northeast, and southwest exhibit higher risk at the beginning of the period. From the mid-1980s to the end of the 1990s, risk increases from north to south in the western and eastern band of the country with regions in the central band of the country keeping lower risk. Then, risks seem to decrease in most regions from 2000 onward, although some provinces remain with high risks, with Castellón the only one with risk significantly greater than one.Figure 3 shows the geographical patterns of colorectal mortality risks for males and females ≥70 years of age. For males, the pattern is quite similar to that for men between 50 and 69 years. There is a risk increase during the whole period in all the provinces. Some northern provinces such as León, Palencia, Valladolid, Vizcaya, Álava, and Guipuzcoa exhibit the highest CRC mortaliy risks in 2008. In general, risks are still growing in nearly all the provinces. For females, the geographical pattern is not so clear. In general, there is an increase in risk until 2004, and then, it starts to decrease. However, there is not a clear gradient from north to south or west to east as it is observed for females aged between 50 and 69 years.

**Table 1:** Colorectal cancer mortality deaths and crude rates (100,000 inhabitants), 1975-2008, by tumour, sex and age group

		**Age**					
			**<50**		**50-69**		**≥70**
		**Cases**	**Crude rate**	**Cases**	**Crude rate**	**Cases**	**Crude rate**
**Males**	Colon	5001	1.05	36144	27.50	71032	136.71
	Rectum	2728	0.57	18861	14.35	32480	62.51
	C+R	7729	1.62	55005	41.85	103512	199.23
**Females**	Colon	4645	1.01	25910	18.02	70932	89.71
	Rectum	2383	0.52	12360	8.59	30022	37.97
	C+R	7028	1.52	38270	26.61	100954	127.69
**Total**	Colon	9646	1.03	62054	22.54	141964	108.35
	Rectum	5111	0.55	31221	11.34	62502	47.70
	C+R	14757	1.57	93275	33.89	204466	156.06

**Figure 4:**
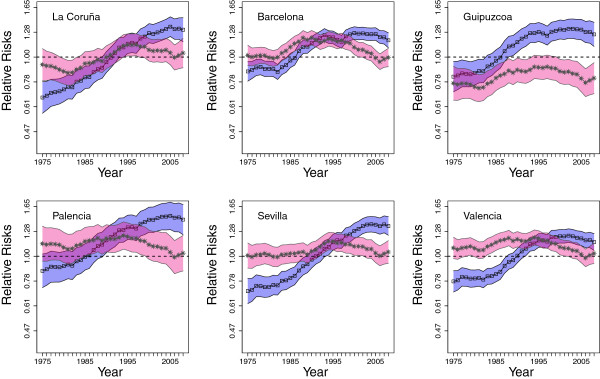
Colorectal cancer mortality risk trends and confidence bands from 1975 to 2008 for males (in blue) and females (in pink) aged between 50 and 69 years.

**Figure 5:**
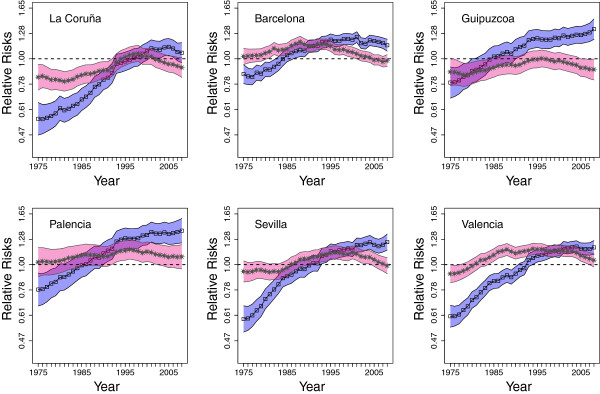
Colorectal cancer mortality risk trends and confidence bands from 1975 to 2008 for males (in blue) and females (in pink) above 69 years.

Figures 4 and 5 display risk temporal trends on a semi-logarithmic scale for six selected Spanish provinces in the period 1975-2008 for age groups 50-69 and ≥ 70 years respectively. These provinces have been selected for illustration purposes. To save space and for better quality of the pictures, temporal trends for all provinces are left as Additional files 1 and 2. In each figure, 95% confidence bands for the risks have also been plotted. Male trends are represented in blue, while the pink ones correspond to females. The provinces have been ordered (from left to right and from top to bottom) according to the geographical location and the Autonomous Region they belong to.Figure 4 portrays colorectal cancer mortality risk for males (blue line-bands) and females (pink line-bands) within age group 50-69 years. As trends are obtained using an indirect standardization method separately by sex and age group, in this figure males and females trends are not directly comparable. Both trends must be compared with one. If the risk (for males or females) in a province and time point is greater than one, it means that the risk is greater than the risk of Spain in the whole period. Hence, the plot represents the evolution of the mortality risk for each age-sex group in the different provinces in comparison to the risk of Spain in the whole period for that age-sex group.

Regarding males, risks are lower than one at the beginning of the study period and they increase with time. From mid-1990s onward risks are significantly higher than one in some provinces and this excess risk does not decrease at the end of the period. Provinces with excess risk are mainly located in the northern strip: the Galician provinces (La Coruña, Lugo, Ourense, and Pontevedra), Asturias, Cantabria, the Basque provinces (Álava, Guipuzcoa, and Vizcaya) and Navarra. In the East, the Catalonian provinces (Lleida, Girona, Barcelona, and Tarragona), Valencia and Baleares islands also exhibit high risks. In the central part of Spain, some provinces of Castilla and León (León, Palencia, Burgos, Valladolid, and Salamanca) display increasing trends with high risks at the end of the period, and finally, the risk is higher than one at the end of the period in some provinces in the South (Cádiz and Sevilla). However, it seems that at the end of the period there is a change in trends in most of these provinces indicating that risks could start to decrease.

For females, trends differ from those for males in the same age group. In general they are rather flat and in most provinces risk for females is not significantly different from that of females in Spain as a whole for the same age group, with the exception of Valencia and Castellón (Mediterranean area) where the risks are significantly higher than one during nearly the entire period. In other provinces such as Lleida, Girona, Barcelona, and León, risk is significantly greater than one during the 1990s. The most striking feature for females is that risk seems to start increasing from 2006 onward after the slight decline observed in the second half of the period. It would be interesting to check if this increase continues in the near future.Temporal risk trends for males and females within the age group ≥70 years are shown in Figure 5. Similar to the previous sex-age groups, trends for males increase with time, and risk is significantly greater than one from the mid-1990s onward in the northern strip, the Mediterranean area (Catalonian and Valencian provinces), central Spain, and the south (Sevilla and Cádiz). A key difference for the age-group 50-69 years is that for males ≥70, the increase in risk persists until the end of the period suggesting that is still growing for most provinces. Trends for females are again rather flat, and the same provinces, Valencia and Castellón, exhibit high risk during almost the whole period. On the other hand, Cuenca, Albacete, Granada, Córdoba, Las Palmas, and Santa Cruz de Tenerife are low-risk provinces throughout the period.

## 4 Discussion

In this study, spatio-temporal patterns of colorectal cancer mortality risks are analyzed for males and females in two age groups. Maps reveal differences by sex. Risk temporal trends by provinces and age groups are also provided. For males in both age groups (50-69 and ≥70), a pronounced increase in risk is observed in the north and central part of Spain, Mediterranean area, and some provinces in the south. For males between 50 and 69 years of age, risks tend to stabilize from 2001 onward, whilst for the age-group ≥70, risks seem to be still growing. A group of high-risk provinces was found in the northwest of Spain. Some of these provinces also have a high gastric cancer mortality risk [31]. For females, the temporal patterns are rather flat along the period, although in age group 50-69 risks seem to increase at the end of the period, a striking feature that requires further research. A clear declining gradient north to south in both the western and eastern band of the country is found in females aged between 50 and 69 years, whereas for females ≥70 years of age, the geographical pattern is not so clear.

A limitation of our study is that it is of ecological nature because we have no explanatory variables related to socioeconomic index, sociocultural habits, or diet. Hence, we can only speculate about the factors that have contributed to the provincial differences observed in the spatio-temporal CRC mortality distribution. Colorectal cancer is believed to be an environmental disease defined by lifestyle factors [7] including diet, physical exercise, tobacco smoking, and use of alcohol [32]. Some studies indicate that dietary factors (such as high red meat intake [33], low vegetable consumption [34] among others) are responsible of 25% of the incident cases [35,36]. Physical inactivity [37] is also associated with an increase of colorectal cancer risk. The Eurobarometer of 2006 indicated that prevalence of physical inactivity in the Spanish adult population was high in comparison with other countries with similar socio-economic level [38]. It is also known that an excess of body mass index (kg/m ^2^) is also consistently associated with high CRC risk [39,40] and some small-area studies have demonstrated that socioeconomic deprivation increases mortality risks of CRC [41]. Temporal trends in this paper reveal different patterns of colorectal cancer mortality risks by sex. It is difficult to explain the different patterns between the two sexes, but these favorable trends in women may be attributed in part to healthier dietary and lifestyle habits. A study that analyzed the principal cancer risk factors in Spain in 2006/2007 reported that the frequency of consumption of fruit and vegetables among the women was higher than in men, 80.7% and 70.9% respectively. The percentage of the obese population (BMI ≥ 30 kg/m ^2^) stood at 15.2% among women and 15.5% among men and the percentage of consumers of alcohol in quantities posing a risk was 1.2% for females (out of 21-40 gr. daily [42]) and 3.3% for males (out of 41-60 gr. daily [42]) in Spain [43]. Very recently, an ecological study was designed to examine the association between colorectal cancer mortality risk and proximity of residence to industrial installations [12]. Those authors suggest that living near industries with pollutant emissions to air could be a risk factor for CRC, detecting higher mortality due to these tumours for both sexes.

The Spanish National Health System is decentralized, with responsibility being delegated to the health systems of the Autonomous Regions. Then, each Autonomous Region is responsible for local application of the cancer screening programs. The Spanish Health Ministry’s Strategic Plan against Cancer [44] following the European Guidelines [45] set up preventive-action programs and guidelines for high-risk groups and planned the implementation of a screening program for medium- to low-risk populations aged between 50 and 69 years, recommending biannual Fecal Occult Blood testing (FOBT) as the first screening option, and leaving each Autonomous Region to decide which specific FOBT should be used (biochemical or immunological) [46,47].

At present, 12 out of 17 Spanish Autonomous Regions have initiated screening programs, and eight of them have results of at least one screening round [48]. The first region initiating a population-based pilot screening program was Cataluña in 2000, followed by Valencia and Murcia over the years 2005-2006. The Basque Country, Cantabria, and the Canary Islands started in 2008-2009, La Rioja in 2010, and Aragón and Castilla-León in 2011-2012. Finally Navarre, Extremadura, and Galicia joined this group in 2013 [13,47]. The remaining regions have undertaken to initiate these programs progressively in the short term [49]. The initiated programs include males and females aged 50-69 years as target population except Cantabria (which starts at age 55 years), Aragón (50-59 for males and 50-54 for females), and Valencia (50-69 for males and 60-69 for females) [13,50]. The data provided by the Spanish Statistical Office showed that the coverage of these programs in Spain was 4% in 2009, 11% in 2010, and 12% in 2011. In 2012 a coverage of 17% was achieved in the whole country. To be precise, 1,744,773 people were included in the programs from a total of 10,283,772 people aged between 50-69 according to the official national census [51]. By regions, the highest coverage was observed in Cantabria (72%), followed by the Basque Country (71%), Aragón (50%), Valencia (46%), Canary Islands (39%), Murcia (28%), and Cataluña (21%). The coverage in the rest of the regions was between 1% and 14%. A 50% coverage is expected in the whole of Spain for 2015 [13].

These programs are relatively new and it is too early to assess their impact on mortality. In the future it will be interesting to examine if there is an association between mortality rates and screening uptake as has happened for breast cancer [52]. Some studies show that the decrease in death is related to the implementation of the screening programs. For example, a reduction in mortality by 16% was achieved after 11 years compared with the neighbouring areas in Burgundy (France) when FOB screening was offered to a population of 90,000 subjects. Incidence of colorectal cancer was unaffected [53].

In conclusion, this updated analysis of spatio-temporal patterns of colorectal cancer mortality in Spain between 1975-2008, divided by sex and age, can offer an interesting picture from an epidemiological and public health perspective. CRC mortality trends show an increase in CRC deaths in the oldest age groups in men. The findings of this paper should be taken into account when deciding whether or not to implement screening programs in all provinces.

## Competing interests

The authors declare that they have no competing interests.

## Authors’ contributions

All authors read and approved the final version of the manuscript and its submission to the journal. JE, MDU, TG, and AFM contributed to the concept and design of the study, to choose and carry out the appropriate statistical analyses, to interpret the results and to write the different sections of the manuscript.

## Supplementary Material

Additional file 1 **Figure S1.** Colorectal cancer mortality risk trends and confidence bands from 1975 to 2008 for males (in blue) and females (in pink) aged between 50 and 69 years for the fifty Spanish provinces. Click here for file

Additional file 2 **Figure S2.** Colorectal cancer mortality risk trends and confidence bands from 1975 to 2008 for males (in blue) and females (in pink) above 69 years for the fifty Spanish provinces. Click here for file
