# Supplementary figures and images for: Age- and sex-specific spatio-temporal patterns of colorectal cancer mortality in Spain (1975-2008)
Source: Popul Health Metr. 2014 Jul 10;12:17. doi: 10.1186/1478-7954-12-17 (PMC4131489; doi:10.1186/1478-7954-12-17)

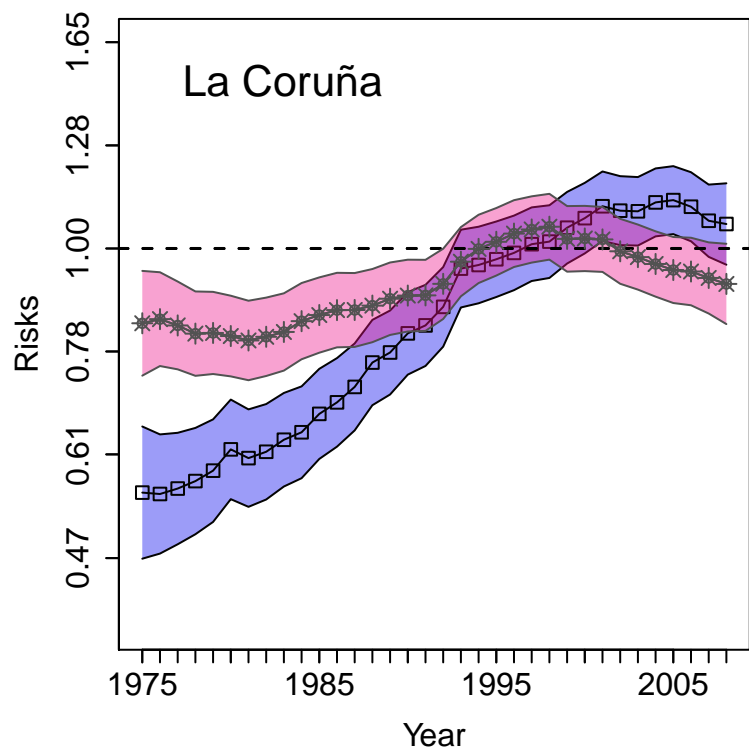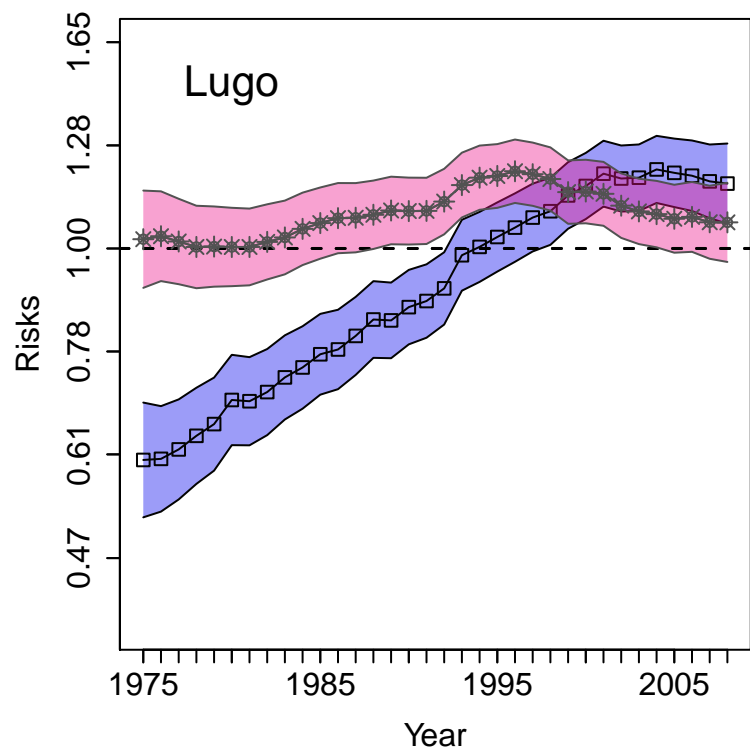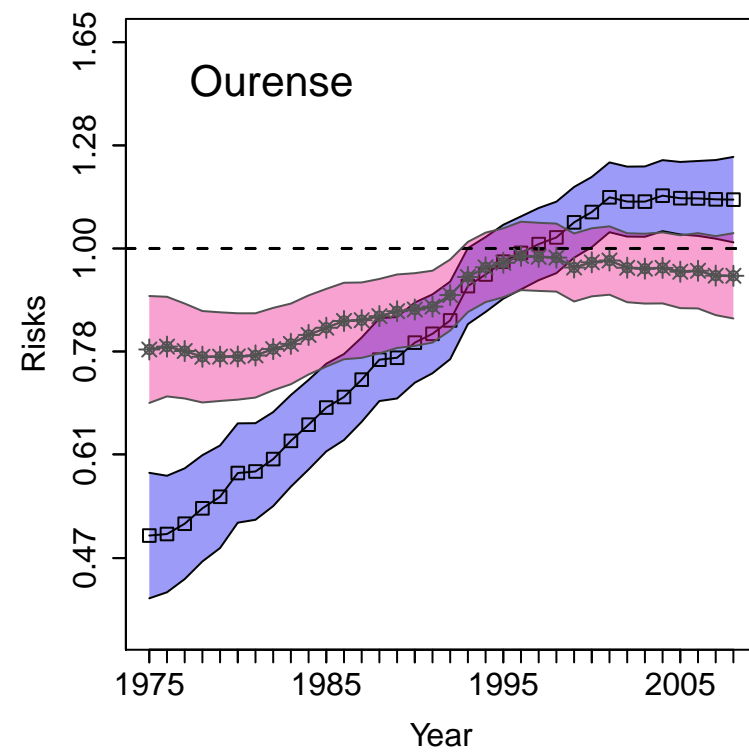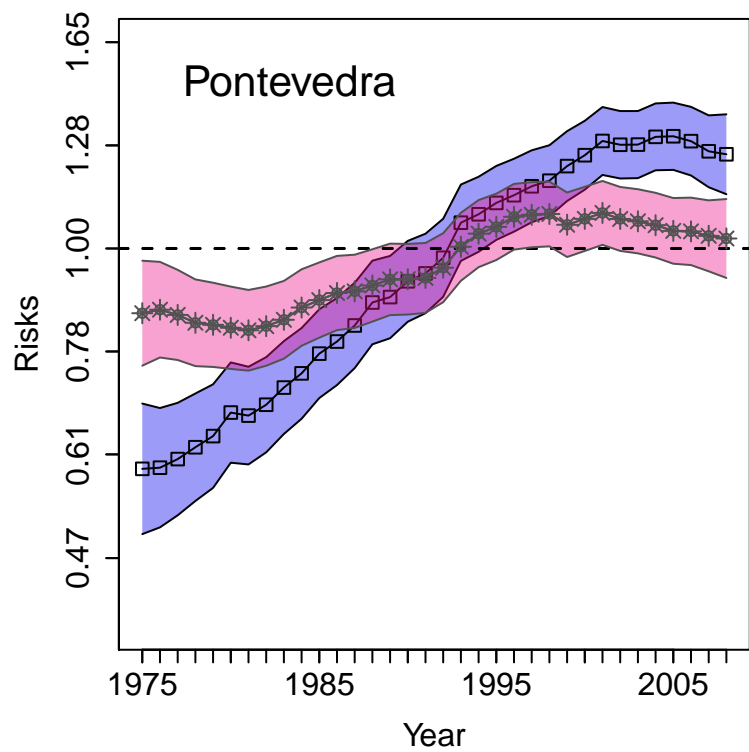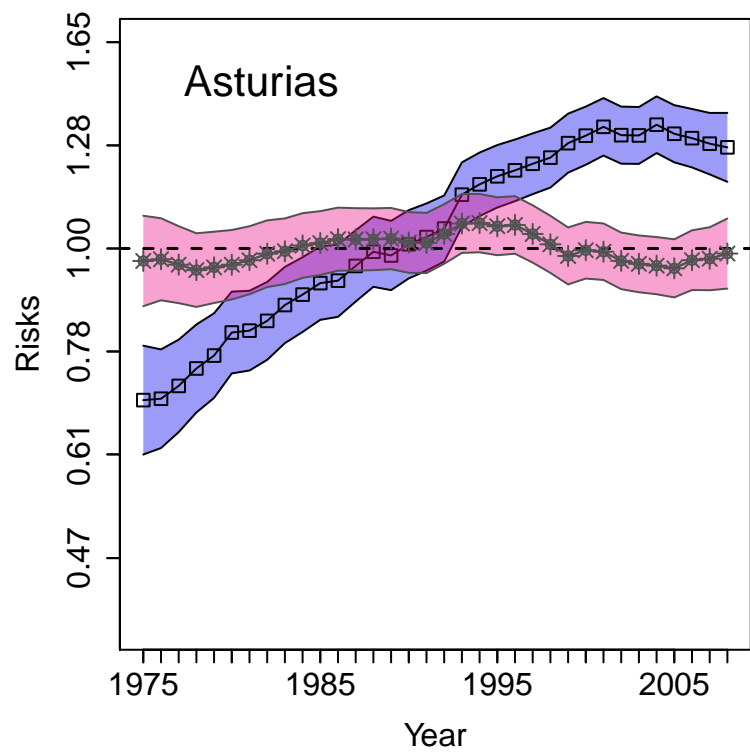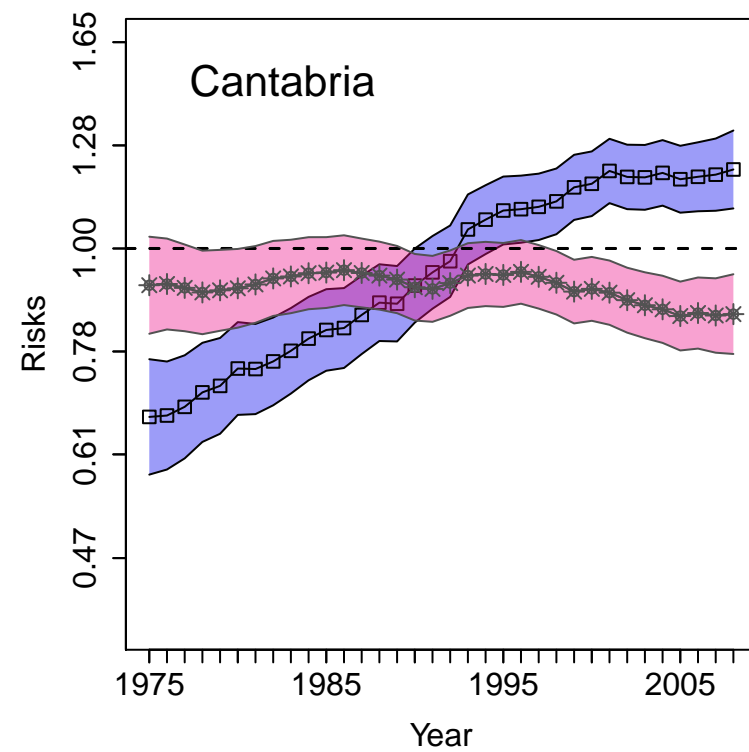

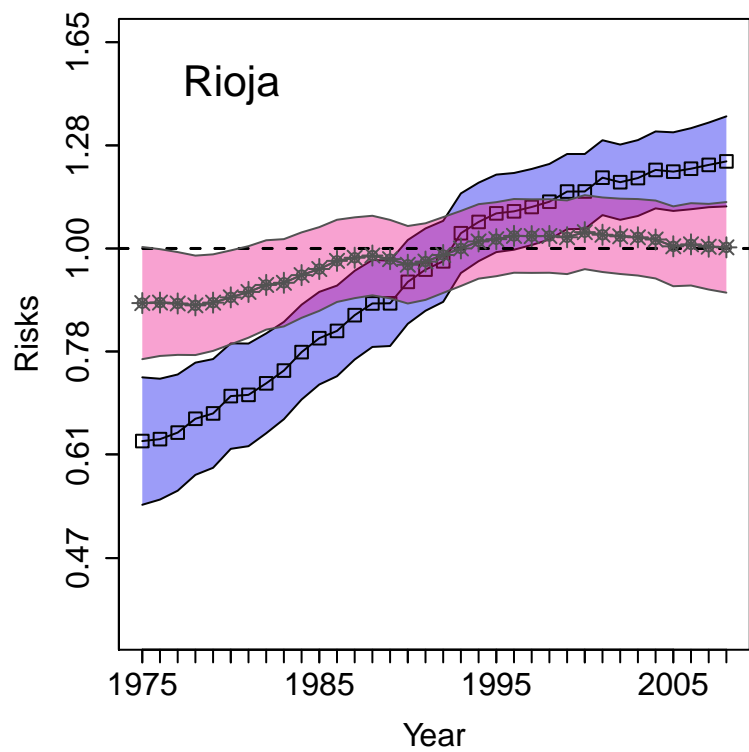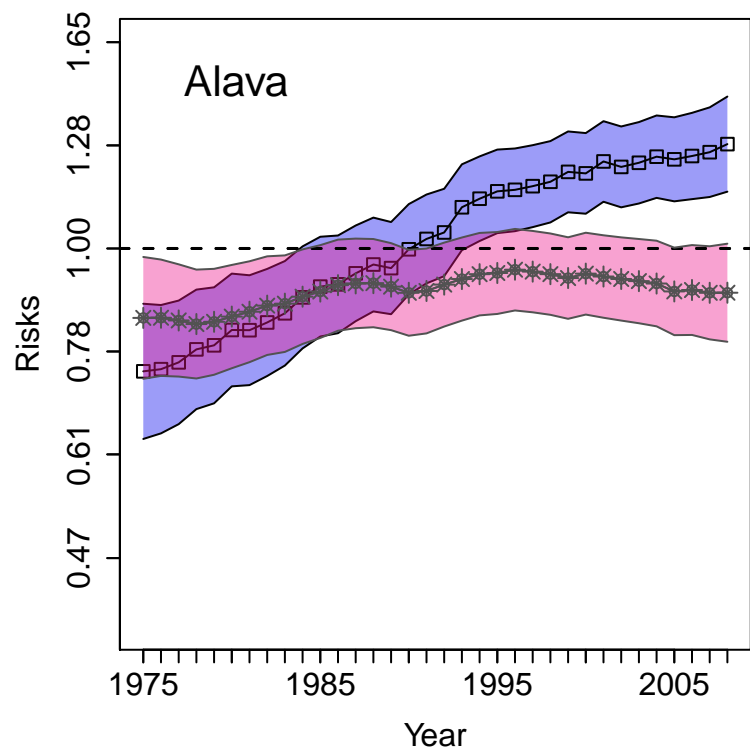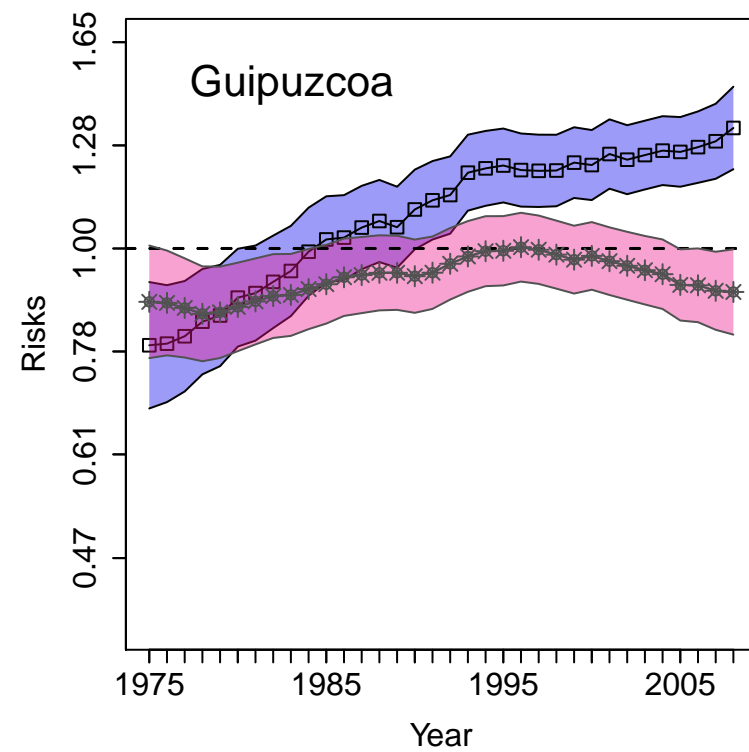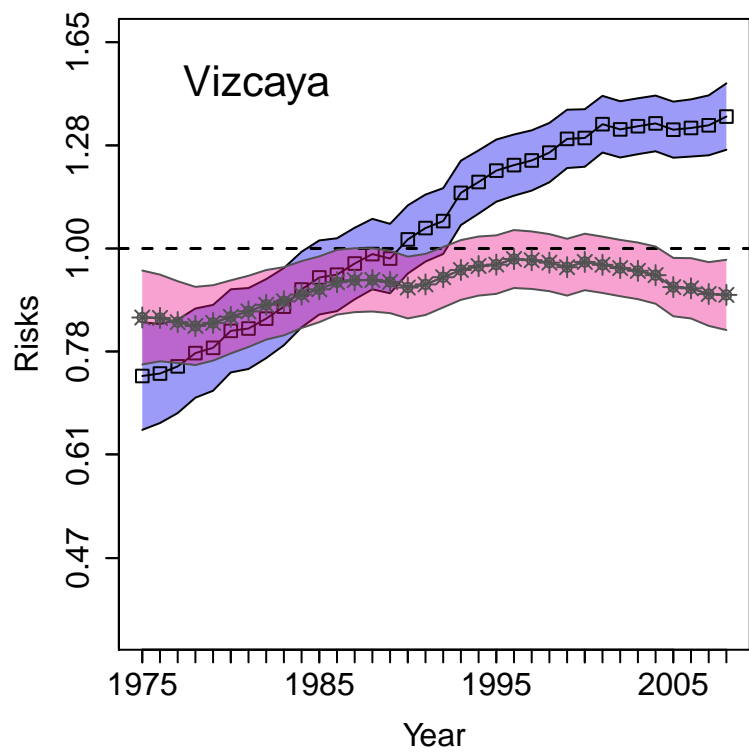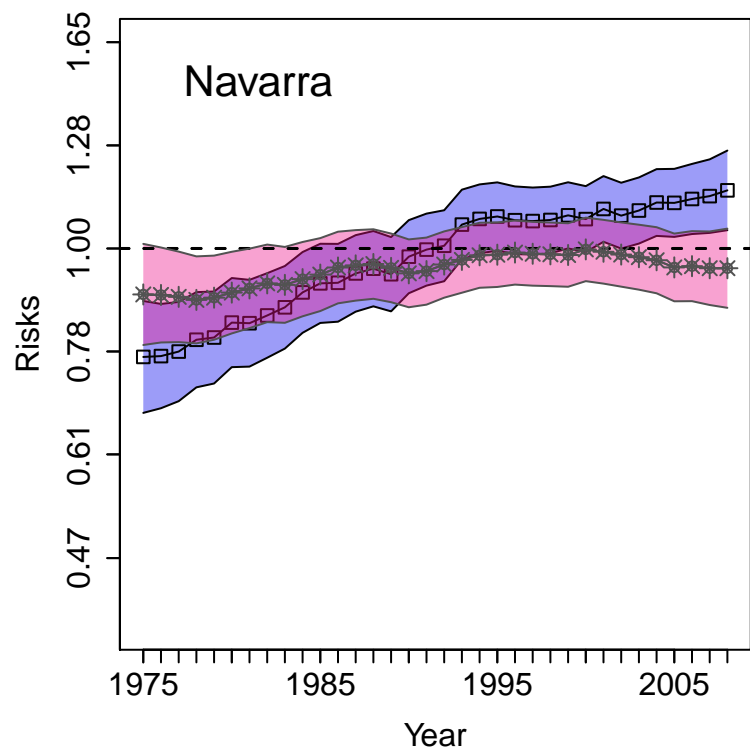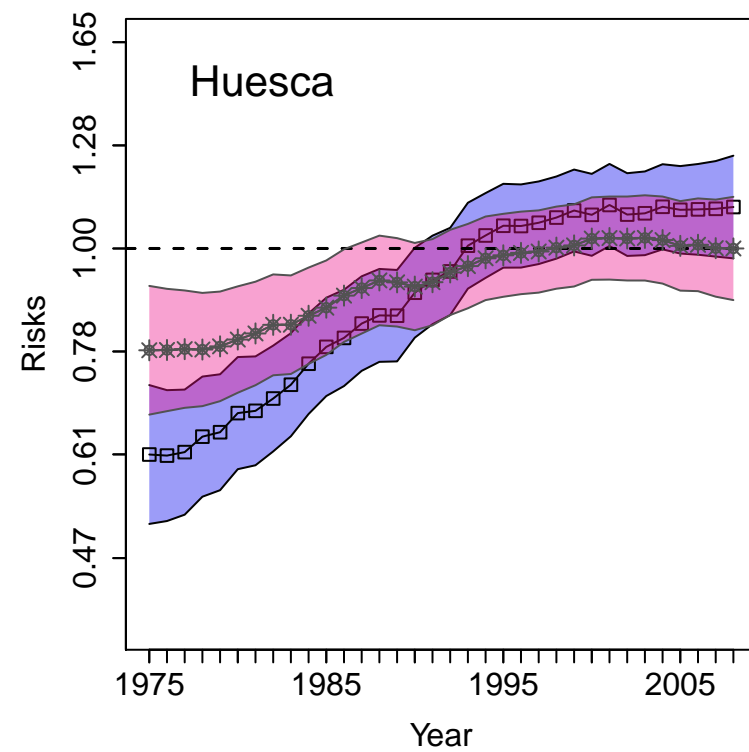

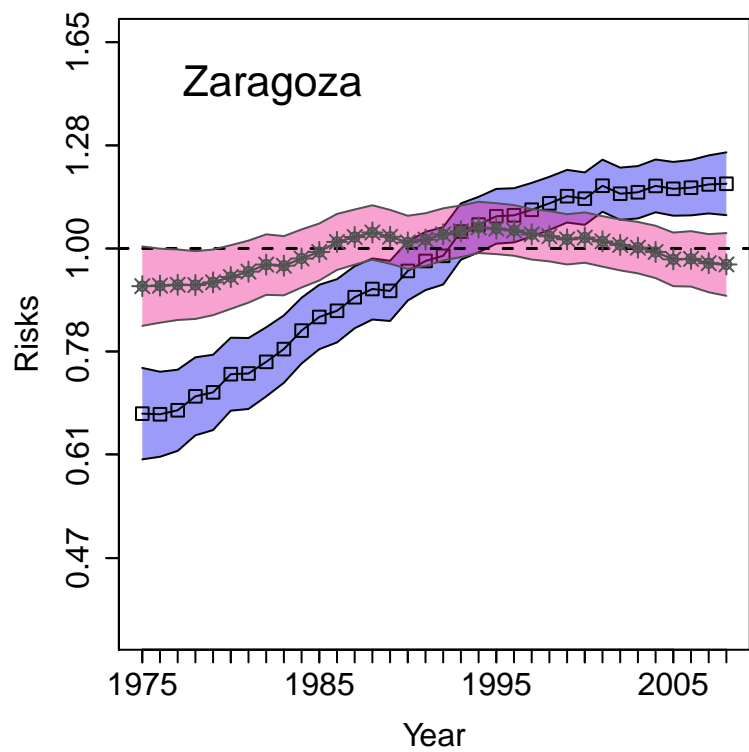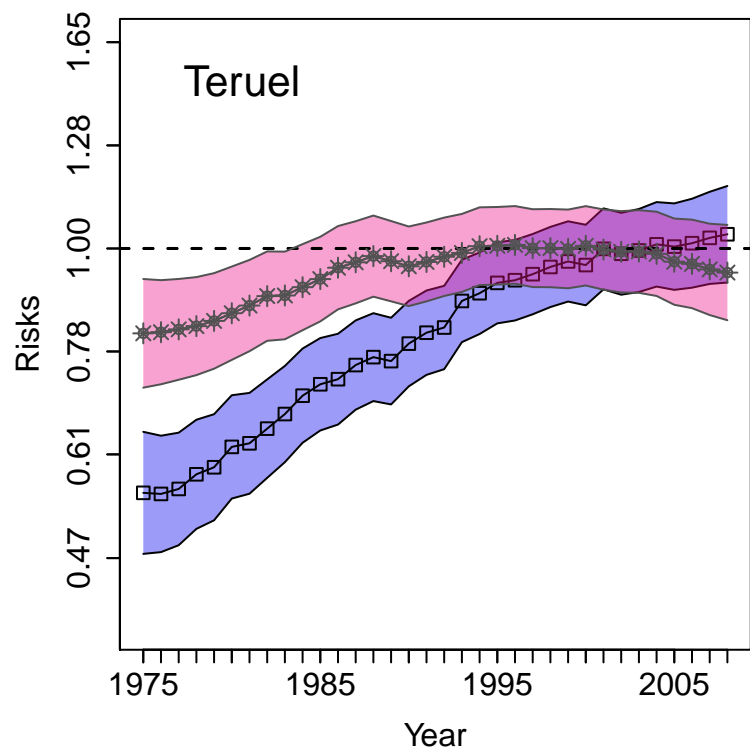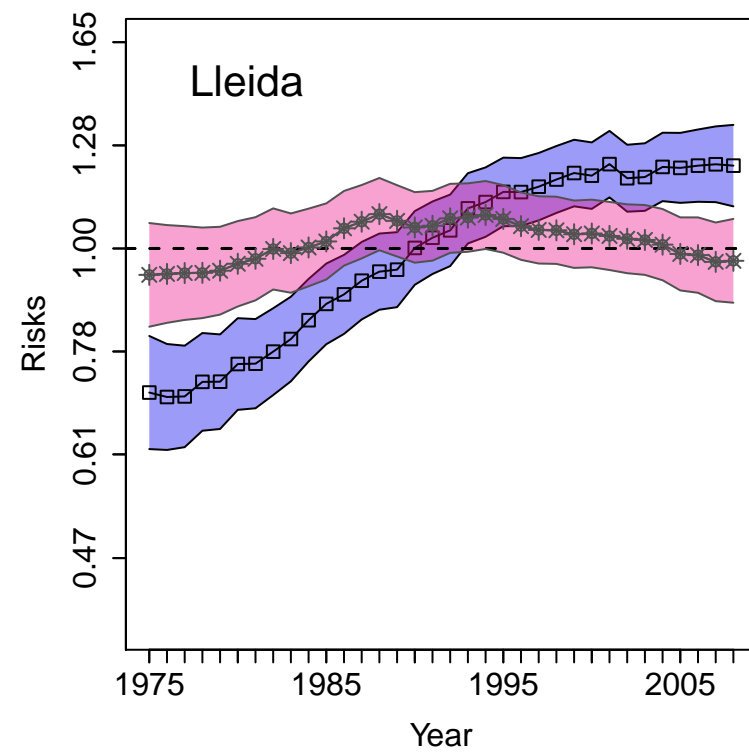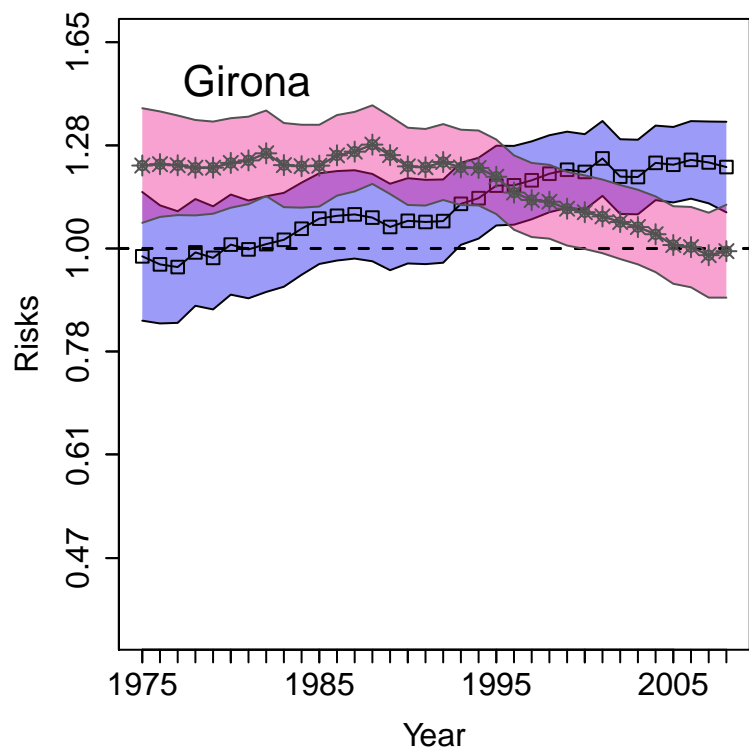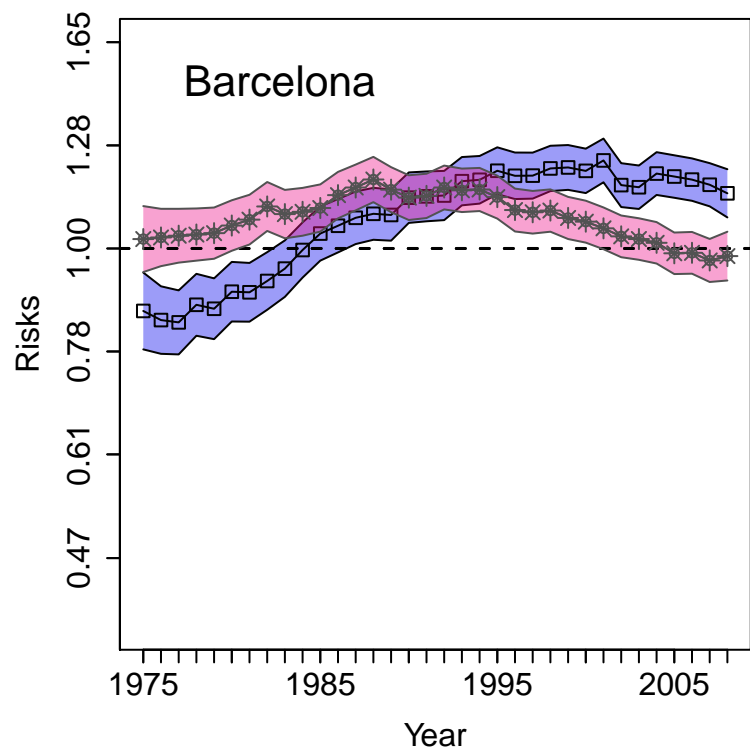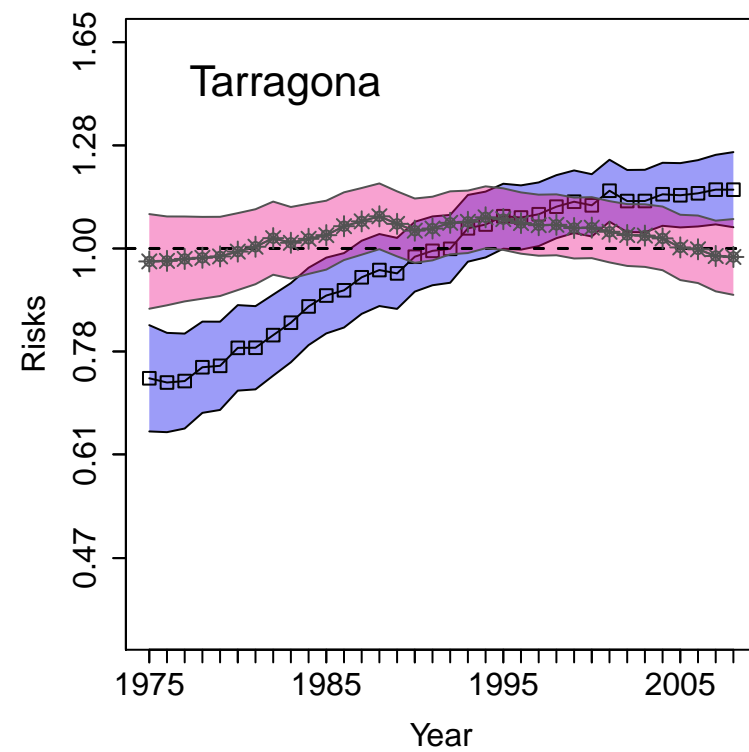

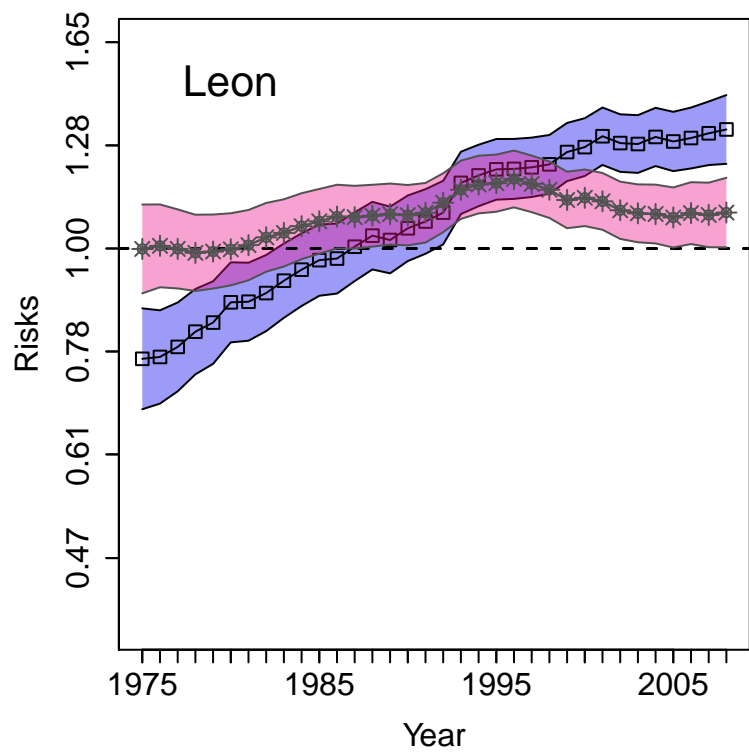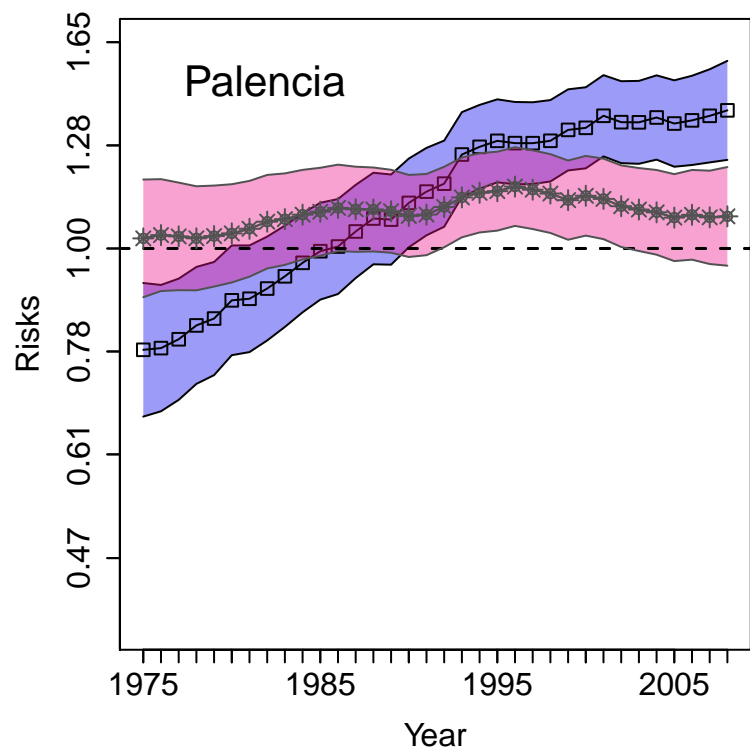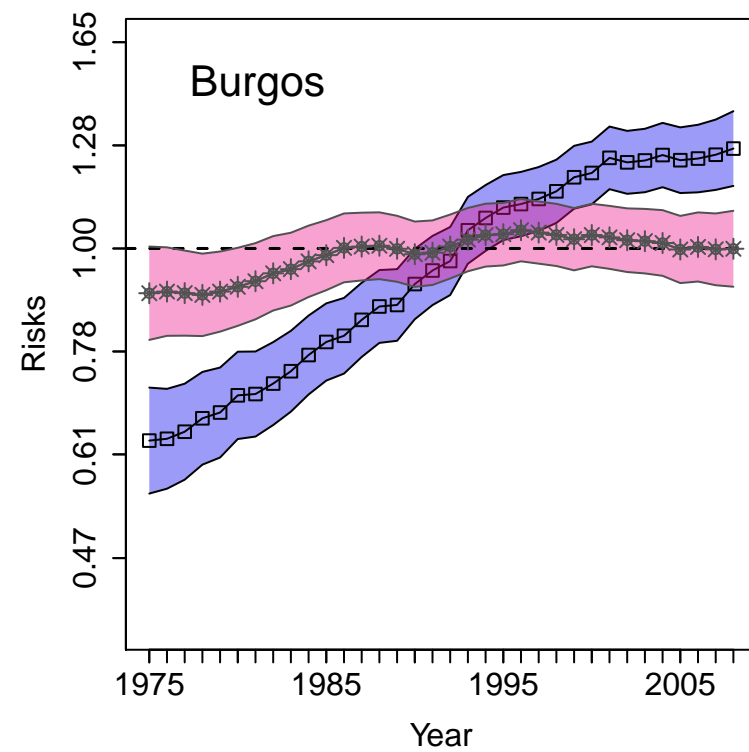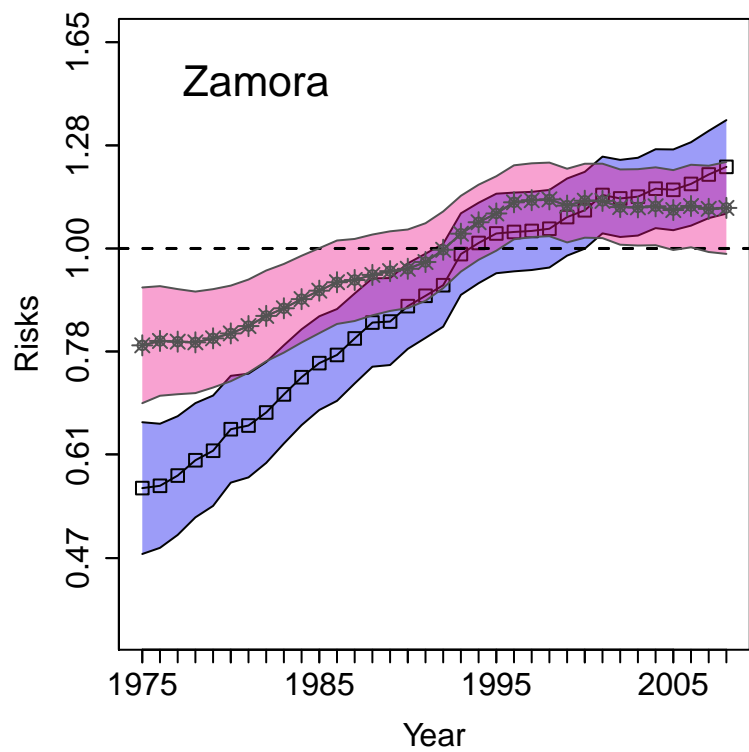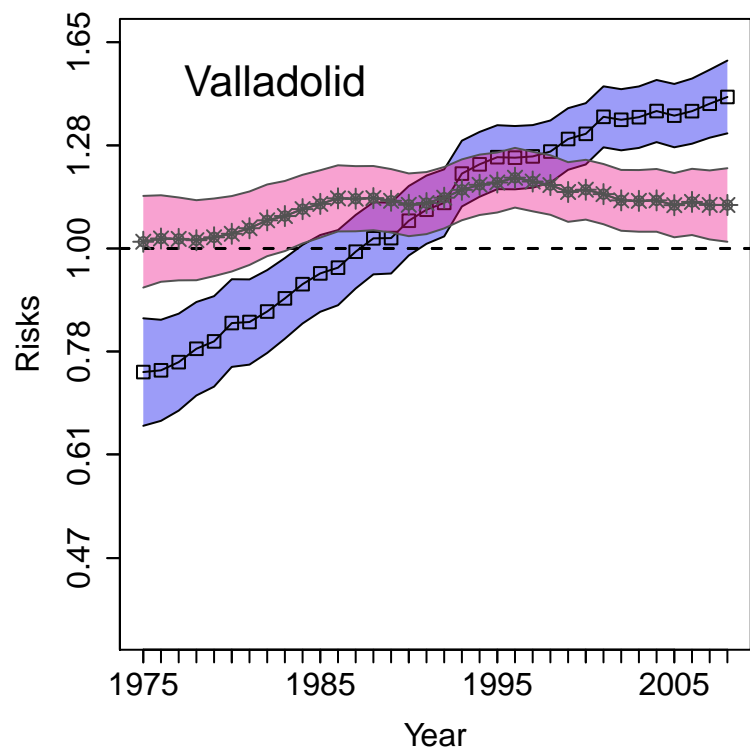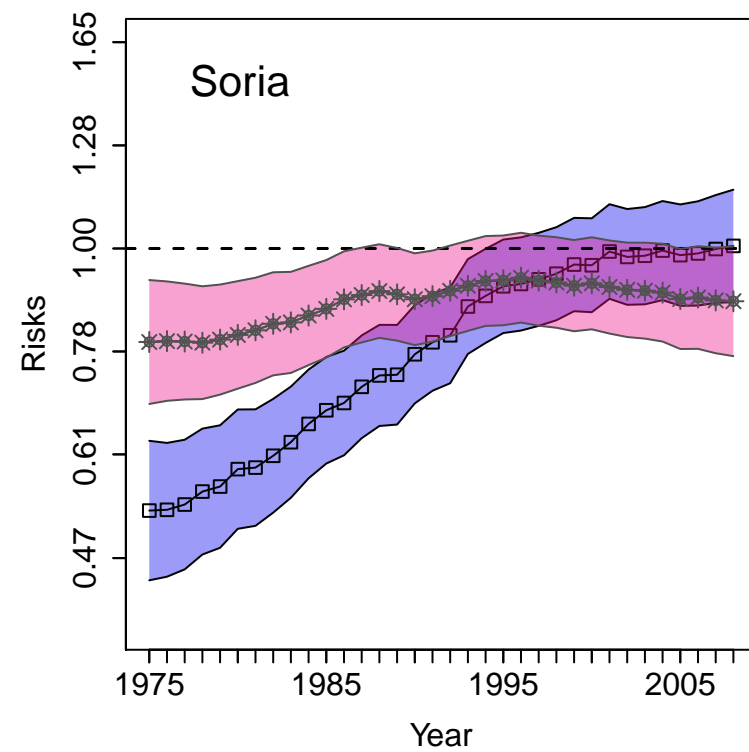

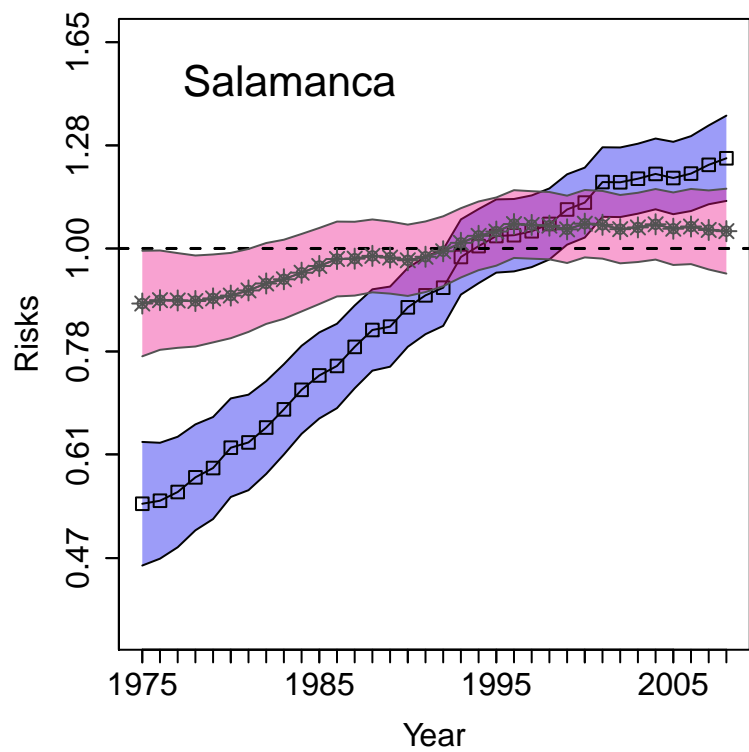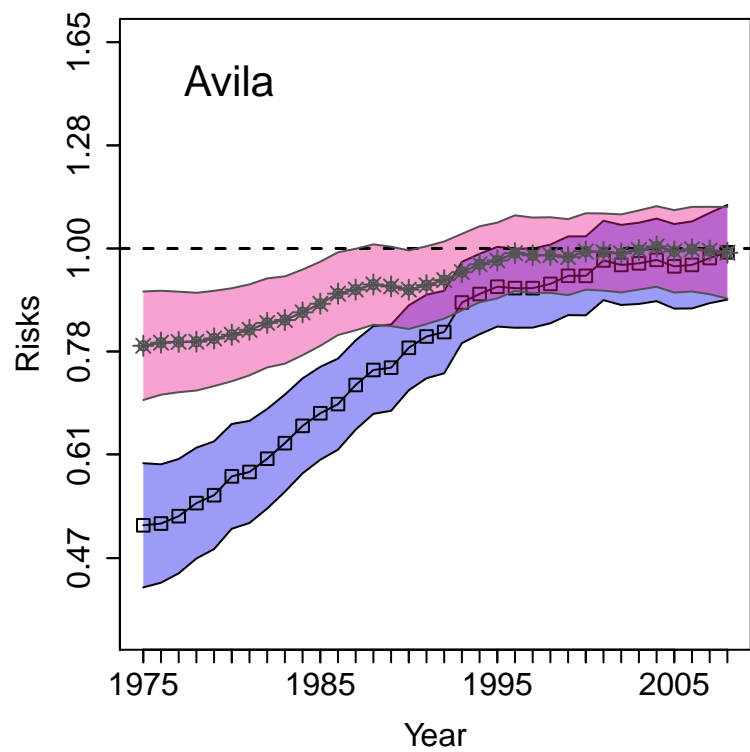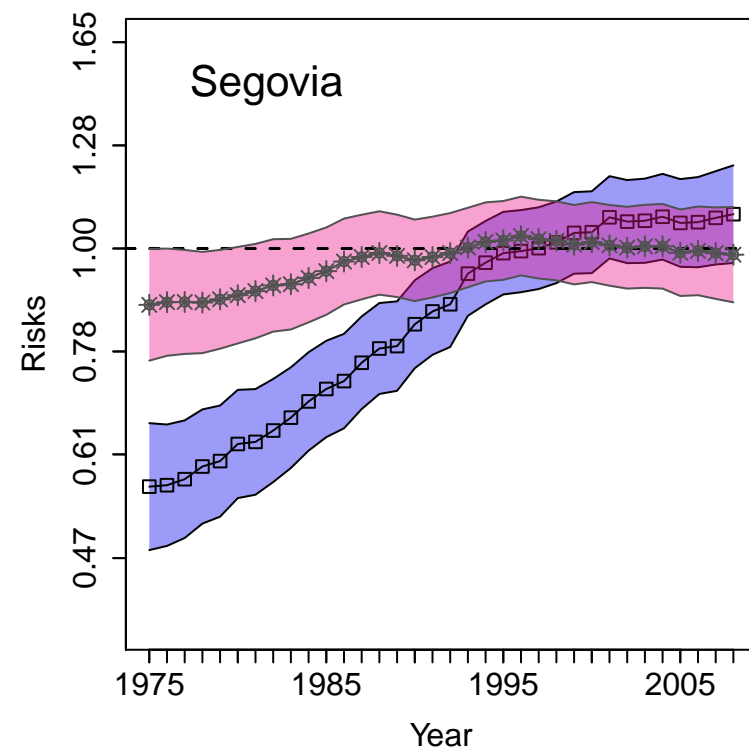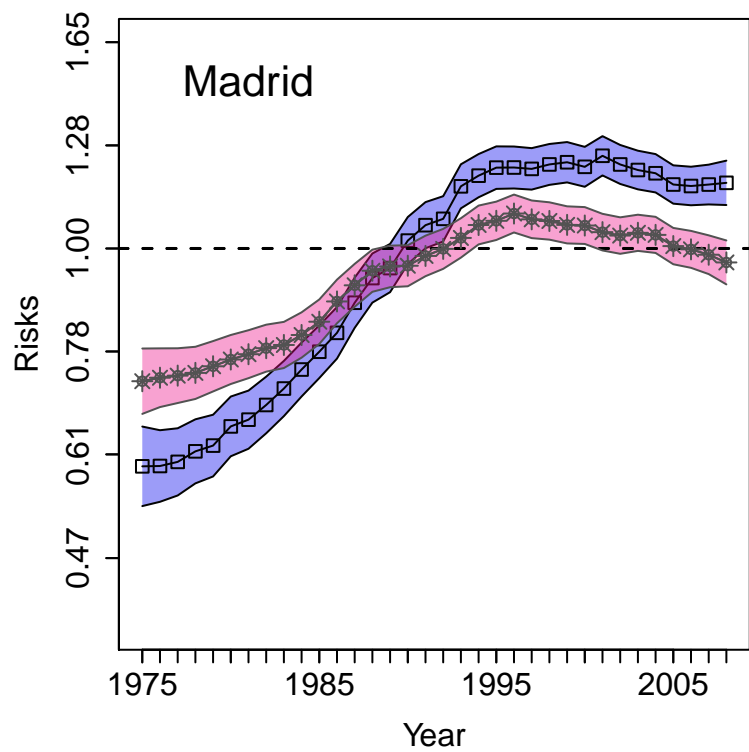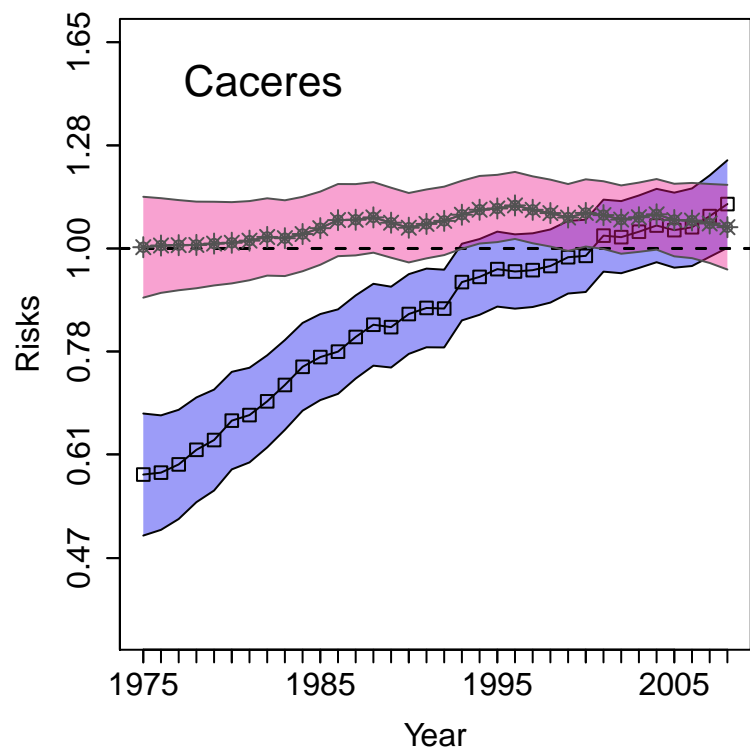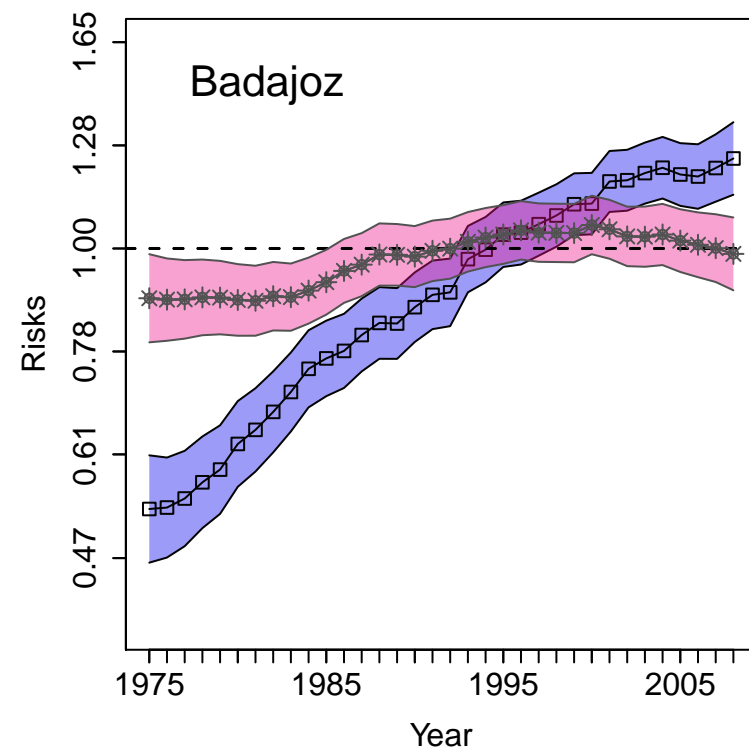

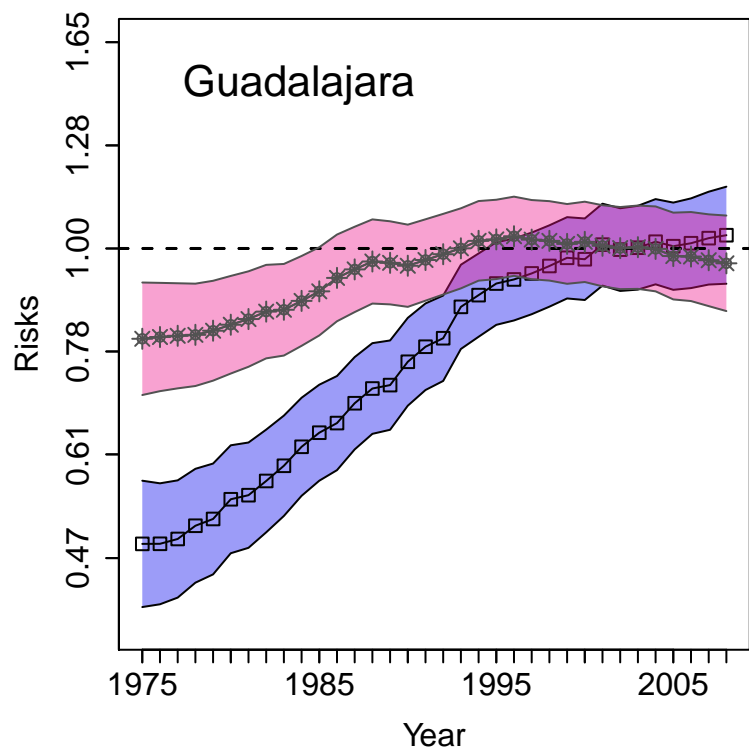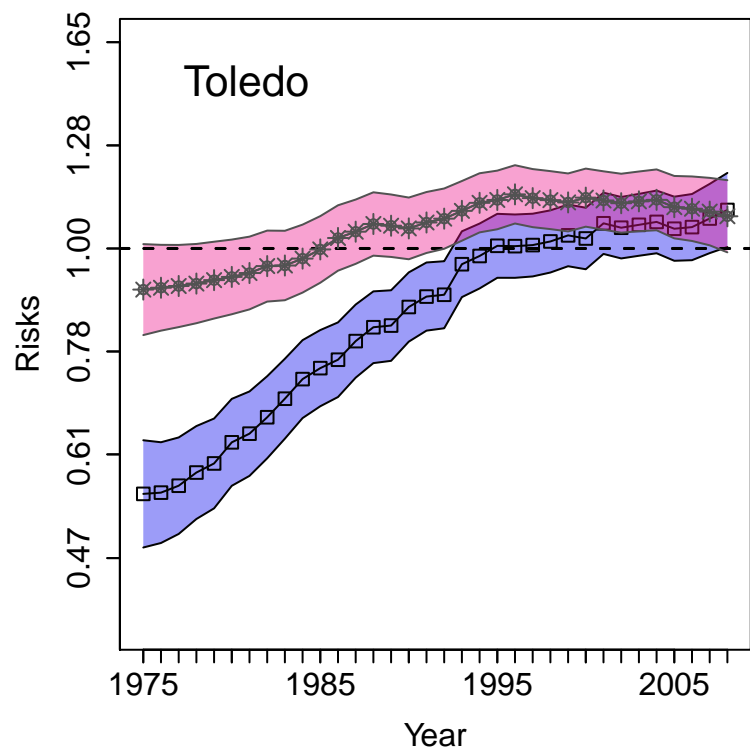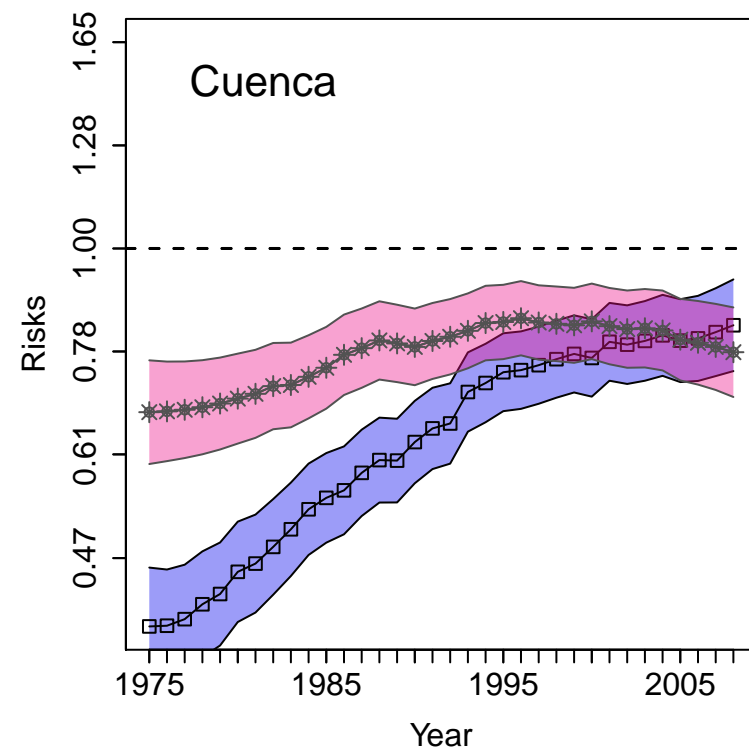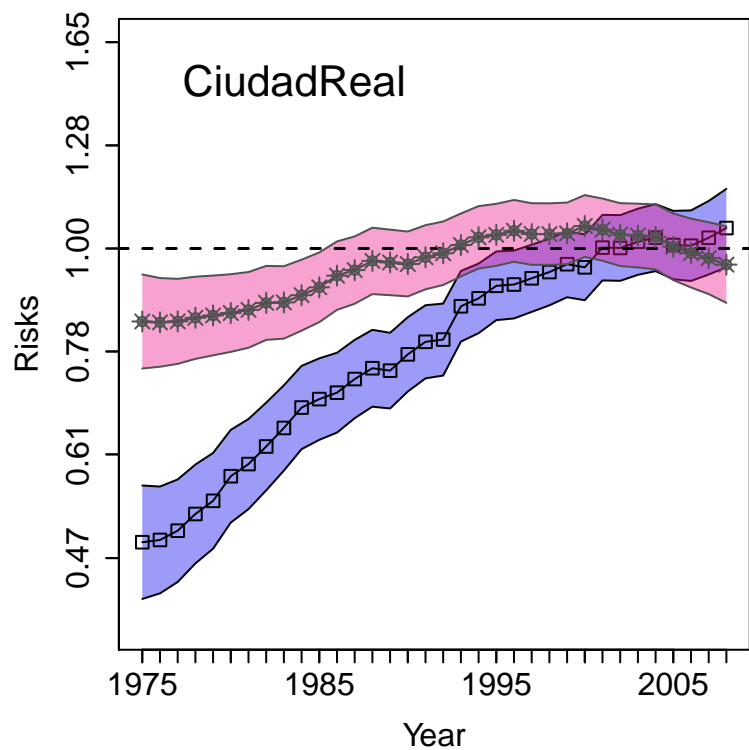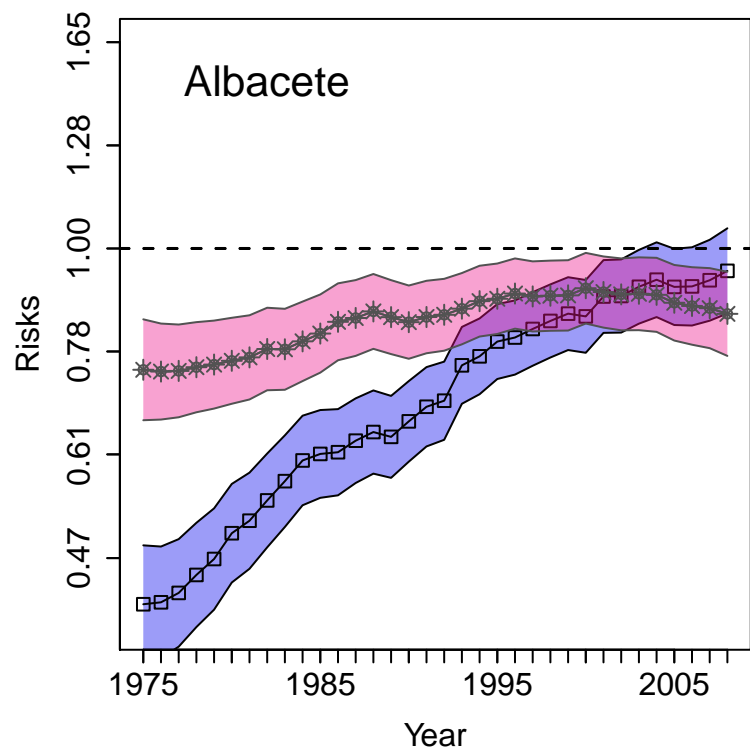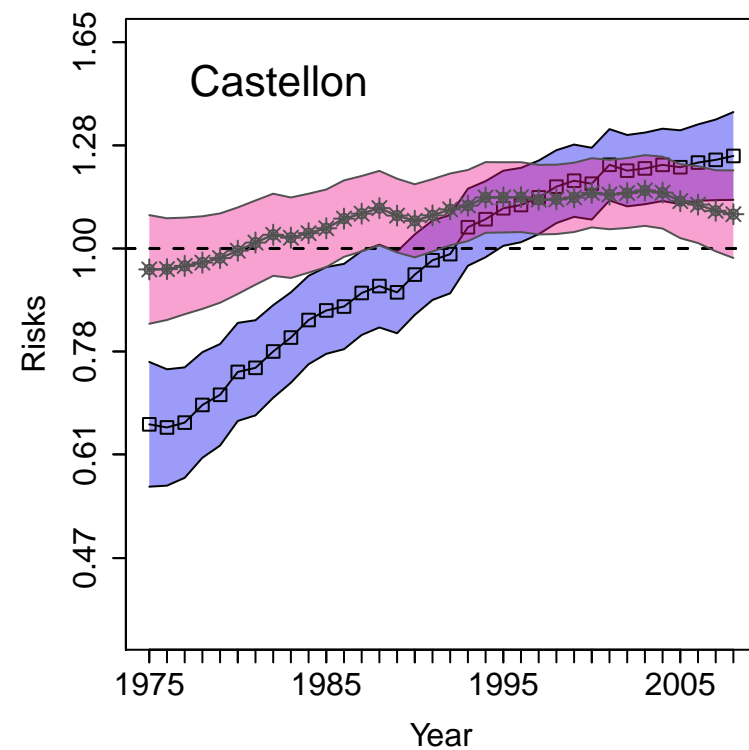

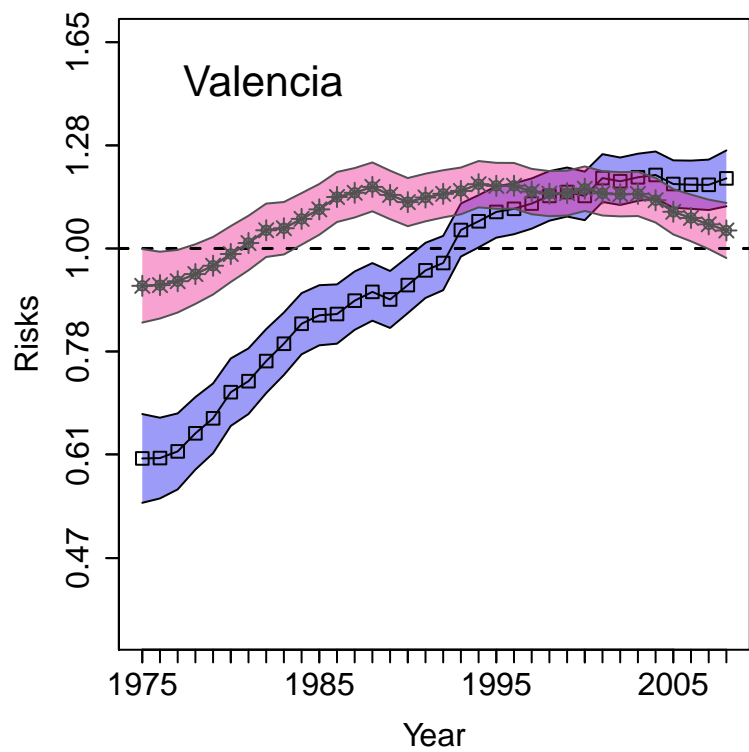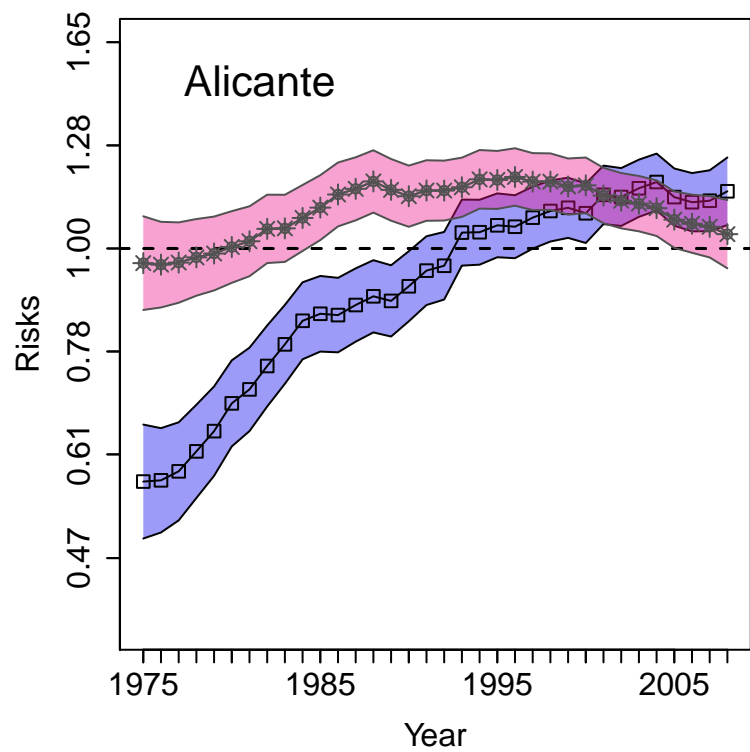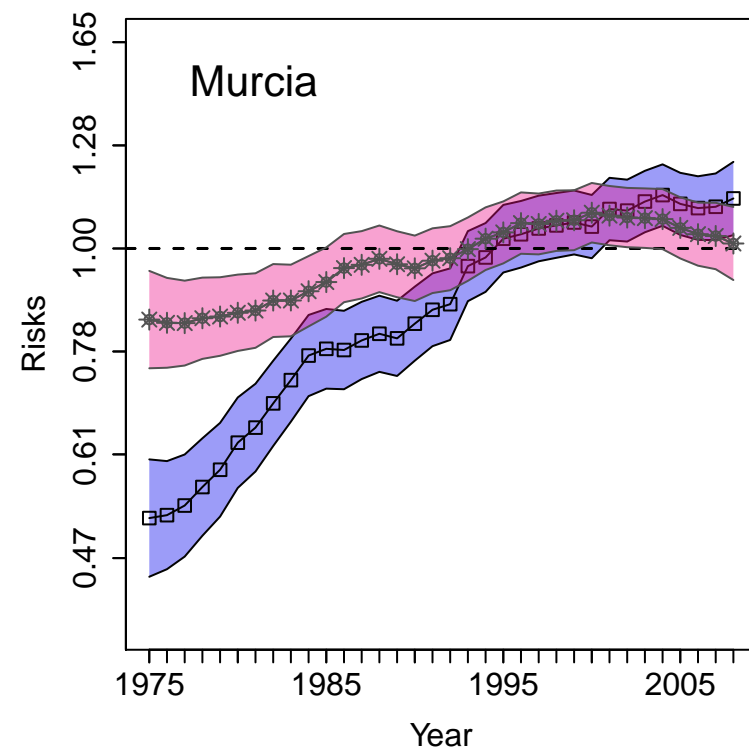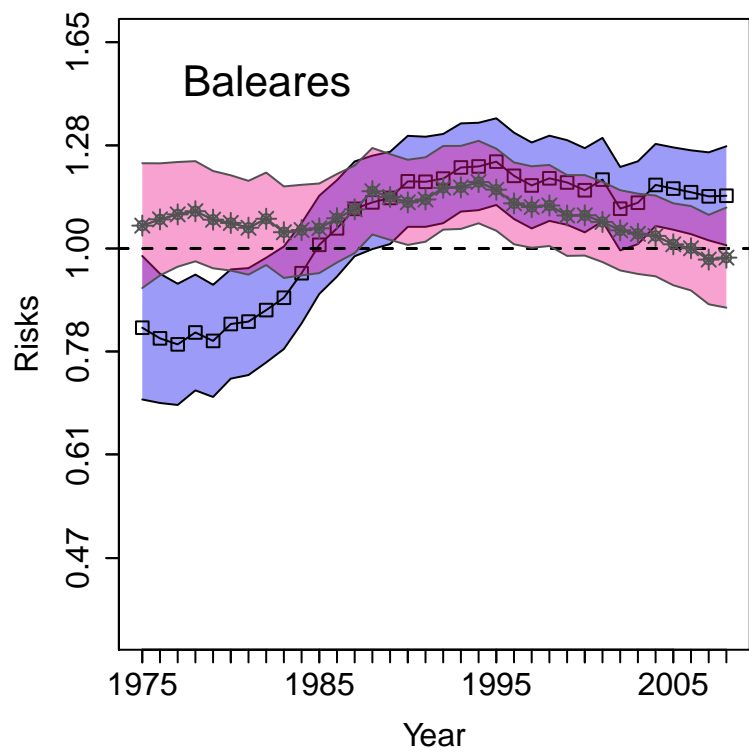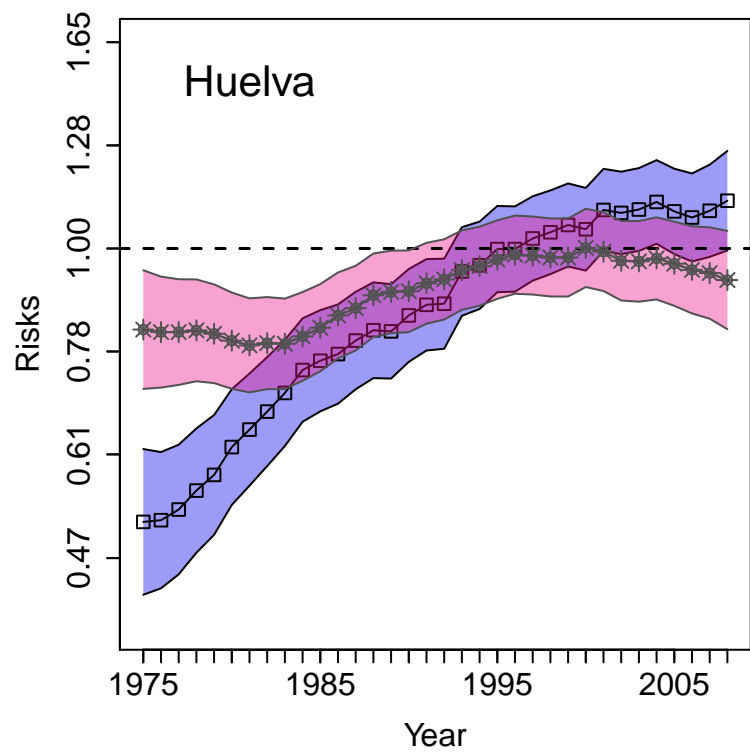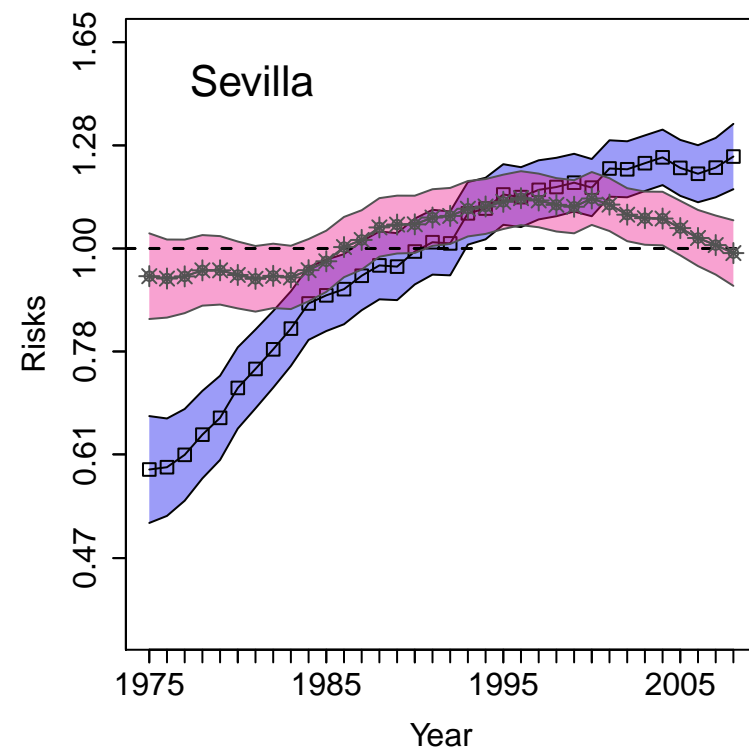

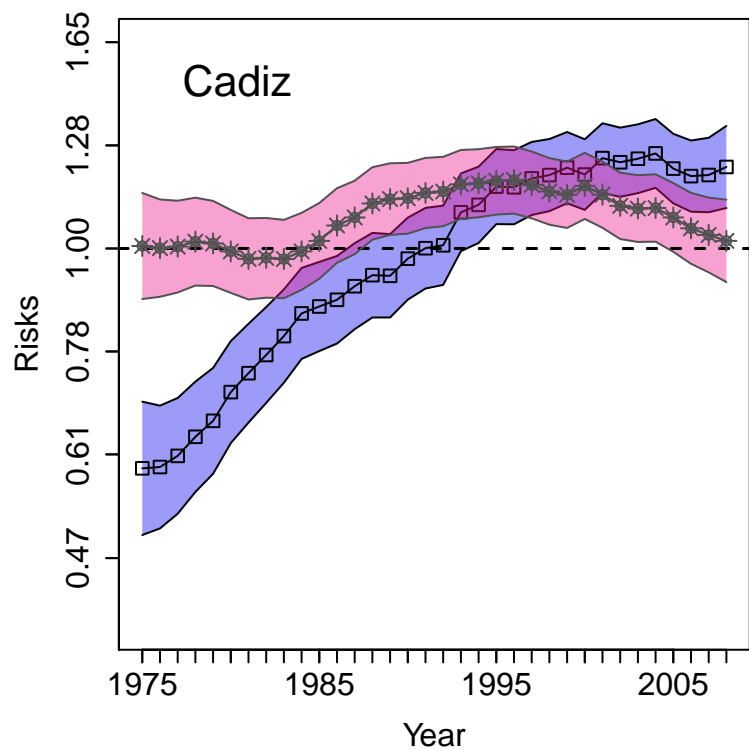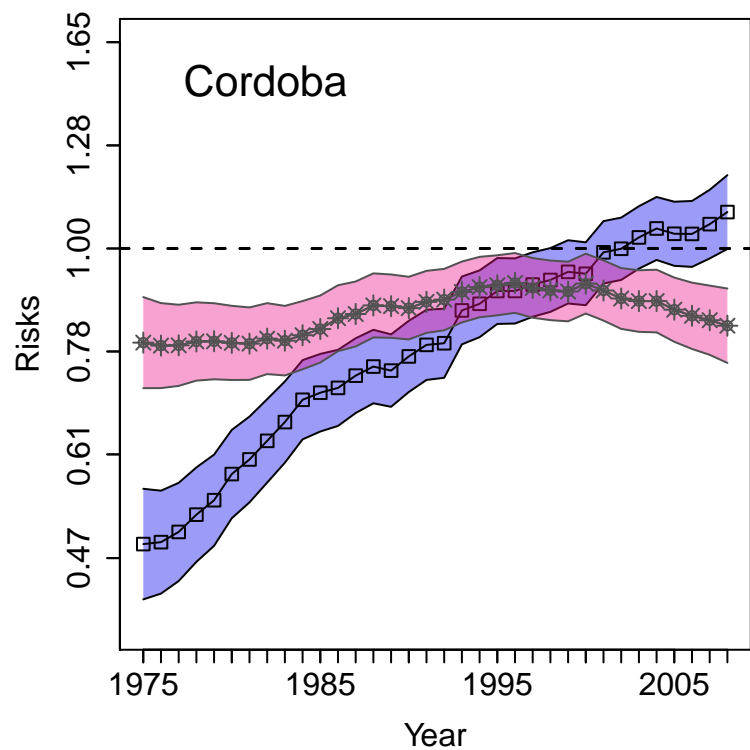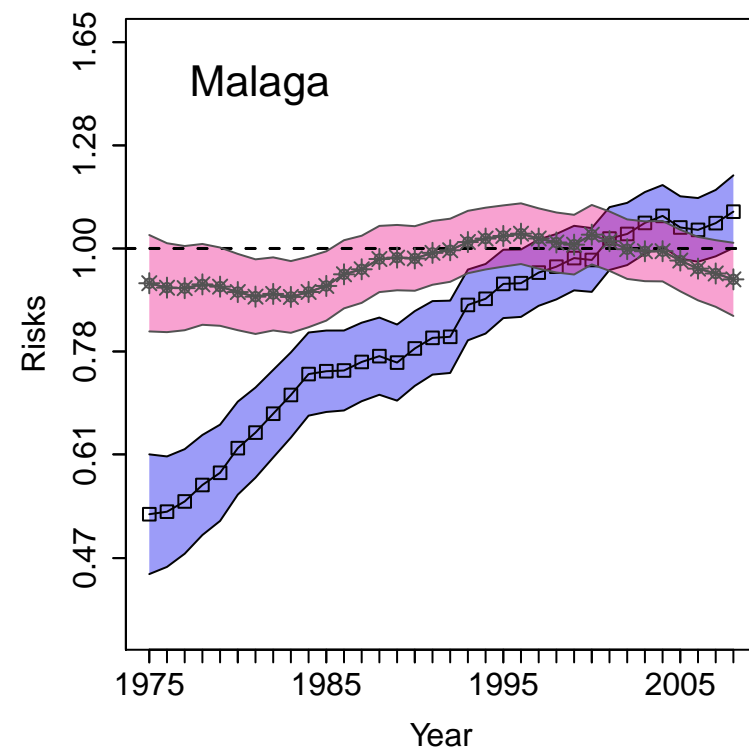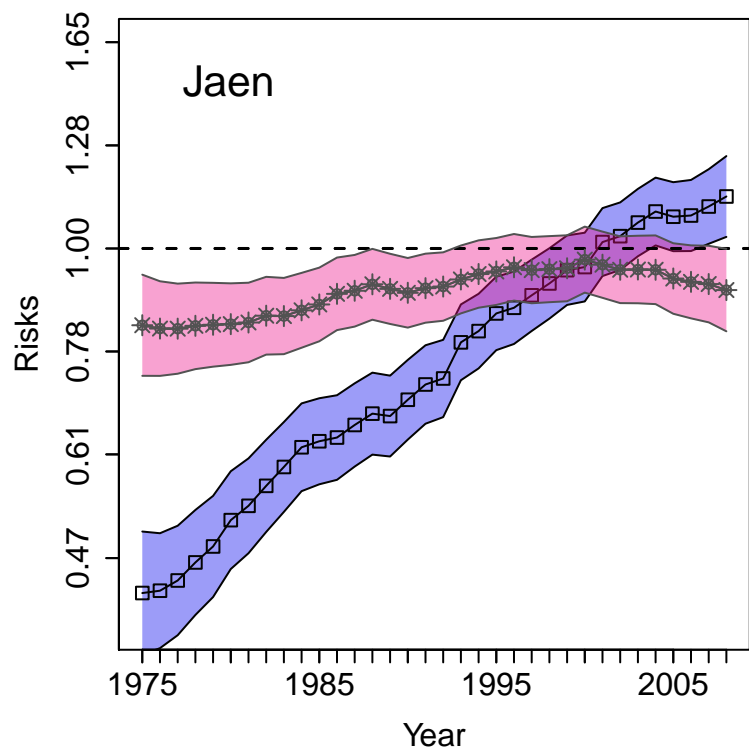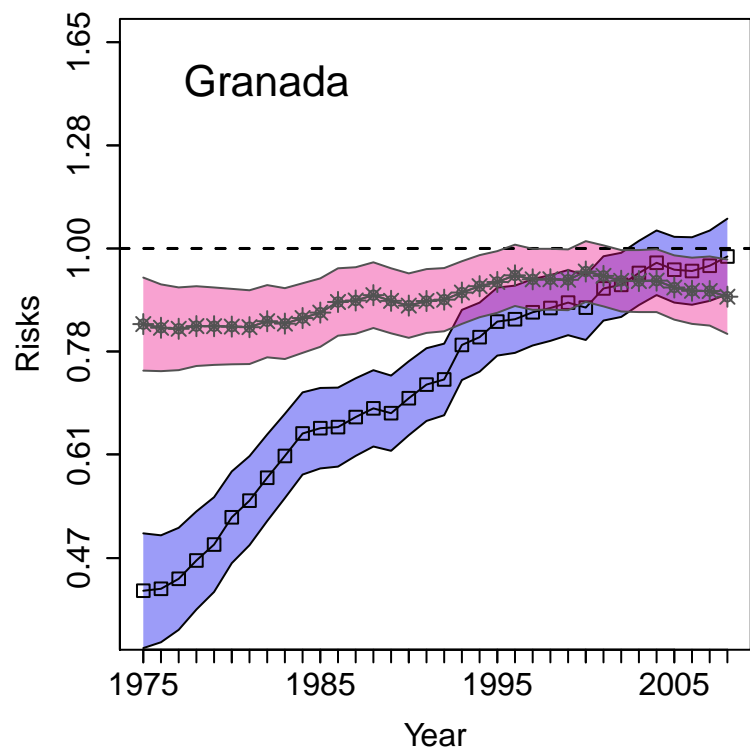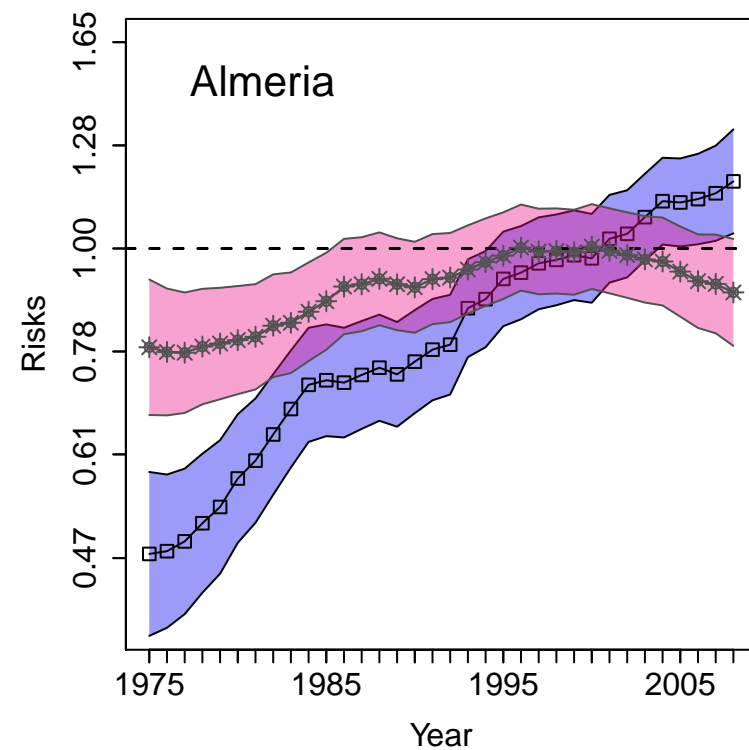

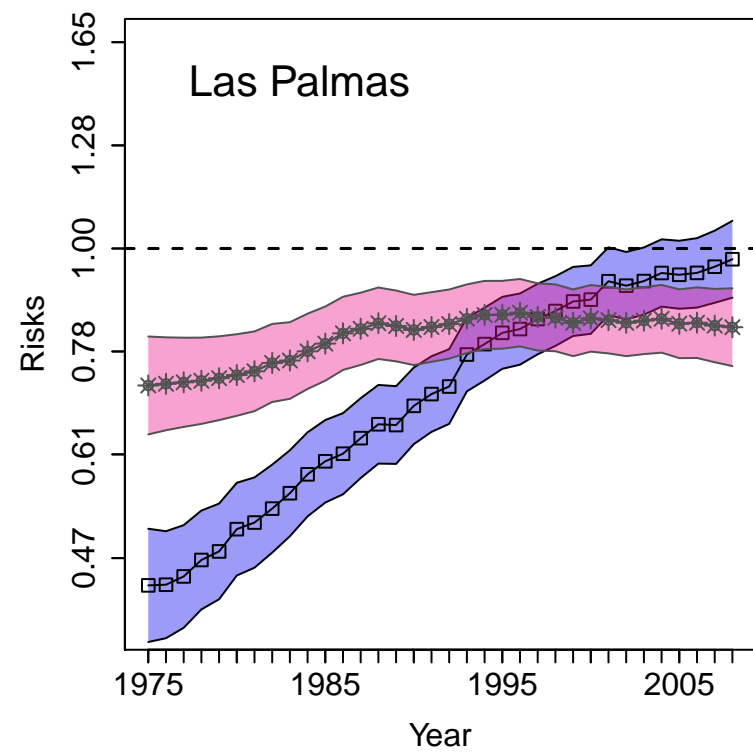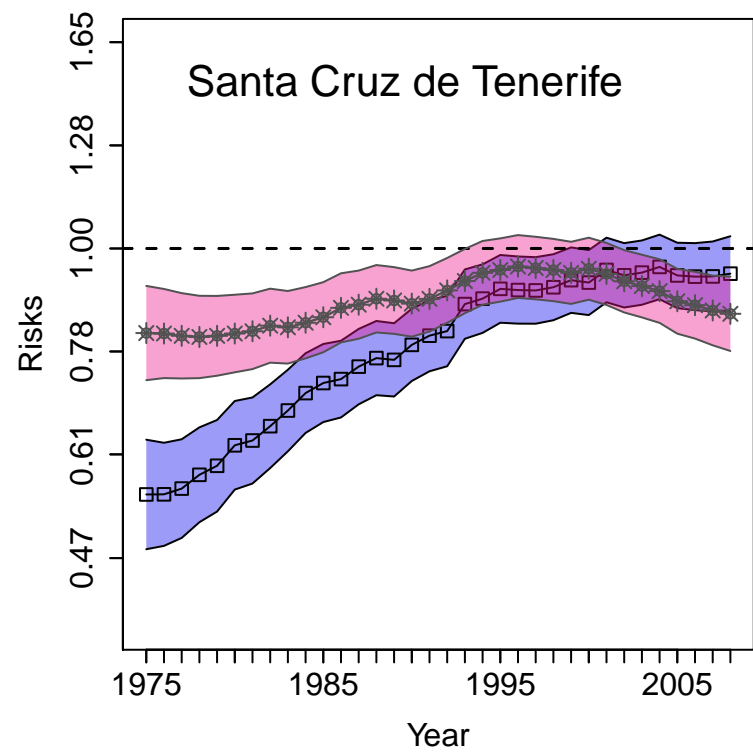

Supplement: Additional file 1 — Figure S1. Colorectal cancer mortality risk trends and confidence bands from 1975 to 2008 for males (in blue) and females (in pink) aged between 50 and 69 years for the fifty Spanish provinces. [file 1478-7954-12-17-S1.pdf]
